# Miniature wireless LED-device for photodynamic-induced cell pyroptosis

**DOI:** 10.1016/j.pdpdt.2024.104209

**Published:** 2024-05-09

**Authors:** Sunghoon Rho, Hailey S. Sanders, Bradley D. Smith, Thomas D. O’Sullivan

**Affiliations:** a Department of Electrical Engineering, University of Notre Dame, Notre Dame, IN 46656, USA; b Department of Chemistry and Biochemistry, University of Notre Dame, Notre Dame, IN 46556, USA

**Keywords:** Photodynamic therapy, Rose Bengal, Wireless LED, Cell death, Pyroptosis, Implantable device

## Abstract

The inability of visible light to penetrate far through biological tissue limits its use for phototherapy and photodiagnosis of deep-tissue sites of disease. This is unfortunate because many visible dyes are excellent photosensitizers and photocatalysts that can induce a wide range of photochemical processes, including photogeneration of reactive oxygen species. One potential solution is to bring the light source closer to the site of disease by using a miniature implantable LED. With this goal in mind, we fabricated a wireless LED-based device (volume of 23 mm^3^) that is powered by RF energy and emits light with a wavelength of 573 nm. It has the capacity to excite the green absorbing dye Rose Bengal, which is an efficient type II photosensitizer. The wireless transfer of RF power is effective even when the device is buried in chicken breast and located 6 cm from the transmitting antenna. The combination of a wireless device as light source and Rose Bengal as photosensitizer was found to induce cell death of cultured HT-29 human colorectal adenocarcinoma cells. Time-dependent generation of protruding bubbles was observed in the photoactivated cells suggesting cell death by light-induced pyroptosis and supporting evidence was gained by cell staining with the fluorescence probes Annexin-V FITC and Propidium Iodide. The results reveal a future path towards a wireless implanted LED-based device that can trigger photodynamic immunogenic cell death in deep-seated cancerous tissue.

## 1. Introduction

Research and development of light-based technologies for diagnosis and clinical treatment continues to expand [1–3]. The most common light-based therapeutic method is photodynamic therapy (PDT), a two-step procedure that first requires administering a photosensitizer (PS) dye to a specific anatomical location followed by illumination of that location [4,5]. Light absorption by the PS triggers an energy transfer cascade that can induce cytotoxic effects and ultimately lead to localized cell death. Typically, these energy transfer processes involve molecular oxygen and generate reactive oxygen species such as excited state singlet oxygen. PDT methods based on short wavelength light are restricted to superficial sites of disease because there is very limited penetration of short wavelength light through skin and tissue. To obviate this clinical limitation, researchers have developed an array of PS dyes and nanoparticles that absorb longer wavelength light which can penetrate more deeply [6]. The inability to utilize short wavelength light for PDT and related phototherapies for deep-seated sites of disease is unfortunate because many blue/green dyes are excellent type II photosensitizers [7], and it is known that cells can be inactivated by different cell death mechanisms that depend on the choice of PS [8,9]. Moreover, the relatively high energy of short wavelength light can activate electron transfer and bond cleavage reactions that are complementary alternatives to the process of energy transfer and singlet oxygen formation [10–12]. These additional photochemical reactions can become the basis of new photoactivated chemotherapeutic methods to inactivate cells and trigger immunogenic responses by pathways that cannot be accessed using PDT [13–15].

Recent research on methods to excite a PS using visible light at a deep tissue location has explored disparate strategies, including X-ray or two-photon excitation of the PS, energy up-conversion using advanced nanomaterials, and implantable light delivery devices [16–18]. The focus of this report is on the latter strategy, namely the use of an implantable wireless, light-emitting device (LED) to excite a PS, an idea that has been described several times in the literature over the last ten years [19–29]. An implantable wireless LED that uses radiofrequency (RF) energy to produce visible light at the site of disease has the potential to circumvent the drawback of limited tissue penetration by the light. In the context of PDT using wireless LED devices powered by RF energy, the published studies can be divided into two groups based on device size, that is, most studies have employed a device with a volume > 50 mm^3^, and only a couple with a volume < 50 mm^3^ [23,29]. The obvious attraction with a smaller device is increased flexibility to employ it at relatively inaccessible anatomical locations and to introduce it by implantation methods that are less invasive. But as the size of an implantable LED device decreases, an important technical question emerges – does the miniature RF-powered device produce enough light to trigger a clinically useful PDT effect? Based on fundamental power laws, the smaller antenna within a highly miniaturized RF-powered LED will result in lower wireless power transfer efficiency and thus inherently lower radiance. On the other hand, healthy cells naturally produce ROS and exploit a range of antioxidant mechanisms to maintain intracellular ROS at a non-toxic basal level [30]. Thus, a miniature LED must produce enough light to ensure that the cytosolic flux of photogenerated ROS is maintained above the basal threshold value for long enough to elicit a clinically meaningful amount of cell death or immunogenicity.

Here we describe the fabrication of a miniature LED-based device with a volume of 23 mm^3^ which is one of the smallest prototypes to date. The miniature LED is powered wirelessly by RF energy and emits light with a wavelength of 573 nm. We have tested its capacity to excite the green absorbing dye Rose Bengal which is a very efficient oxygen PS and has potential for use in wide range of therapeutic areas [8,31–34]. In the case of cancer, PDT using Rose Bengal is known to induce a long-lasting cell-killing effect that proceeds over time by multiple pathways and releases immunogenic molecular signals [8,35,36]. We find that the combination of miniature wireless LED device as light source and Rose Bengal as PS can photogenerate enough singlet oxygen to induce cell death within monolayer cultures of HT-29 human colorectal adenocarcinoma cells. Furthermore, we report evidence that a major cell death pathway is pyroptosis and we discuss the potential of this photodynamic technology to eradicate deep-seated tumors in a living subject by triggering an immunogenic response.

## 2. Materials and methods

### 2.1. Fabrication of miniature wirelessly powered implantable led device

**Fig. 1.**
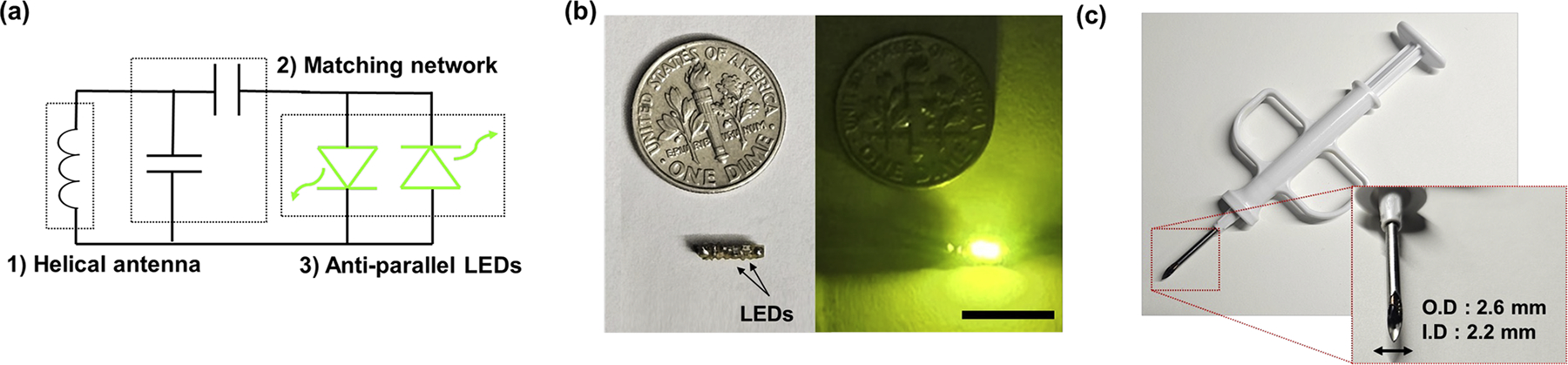
(a) Schematic of the LED device. (b) Comparison of the device with a US dime (scale bar: 10 mm). (c) The device fits within a 12 G biopsy needle.

The device is composed of a printed circuit board (PCB), a helical copper-wound, ferrite-core antenna (diameter 1 mm), two green LEDs (573 nm, Wurth Elektronik, 155060VS73200), and an impedance matching network as shown in Fig. 1a. The dimensions are 1.5 × 7.4 × 2 mm and the volume is 23 mm^3^ (Fig. 1b) which is small enough for injection into biological tissue using a 12 G biopsy needle (Fig. 1c). The detailed fabrication procedure followed the same steps described in our previous work [37]. Circuit components, including a simple matching circuit with two 0201-sized capacitors, were manually soldered onto the 0.4 mm thick PCB board, and an anti-parallel LED configuration was applied for rectifying AC to DC instead of using a separate rectifier. The impedance matching network was designed to operate at the 6.78 MHz industrial, scientific, and medical (ISM) band for biomedical applications. Finally, the entire device was coated with biocompatible epoxy (EpoTek, MED-301) to provide durability and biocompatibility.

### 2.2. Wireless powering system

The wireless powering system consisted of a 6.78 MHz transmitter (Wibotic TR-301 controller) set to continuous wave, constant power emission up to 6 W. The dimensions of the transmitting antenna (Wibotic TC-200-HP-ST) were 19 cm × 19 cm × 2 cm. The device evaluation and cell culture photoinactivation experiments used 2 W, 4 W or 6 W of transmitting RF power.

### 2.3. Optical output measurements

The radiant power of the wirelessly powered device was measured using an external silicon photodiode (Thorlabs FDS1010) that was placed directly next to the device. Additionally, two linear translational stages were employed to measure the optical output of the device as a function of transmitter distance, using 3D-printed holders to precisely position the components. The photocurrent generated by the LED device illumination of a large area photodiode was measured using an ammeter (Keithley, 2450 Source meter). The photocurrent was converted to incident optical power using the photodiode’s responsivity at the peak LED emission wavelength. To mitigate the effects of ambient light, all light sources were turned off during the measurement and dark current was subtracted from the measurement.

### 2.4. Singlet oxygen measurements

Stock solutions of 1,3-diphenylisobenzofuran (DPBF) and Rose Bengal were prepared in methanol and used to prepare a 1 mL methanol solution of DPBF (75 μM) and Rose Bengal (6 μM) in a quartz cuvette. The cuvette was inverted 5 times and left exposed to the atmosphere during irradiation with the device located inside the cuvette and located 1 cm away from the transmitting antenna. The device was wirelessly powered using different transmitting RF power levels (2 W, 4 W, and 6 W) and the solution was irradiated for a total of 180 s. After every 30-second interval, an absorbance spectrum (300 – 600 nm) was recorded. The decrease in DPBF absorbance at 415 nm reflected the amount of singlet oxygen that was photogenerated. The laboratory lights were off during the entire experiment to minimize ambient light interference.

### 2.5. Cell culture conditions

HT-29 (ATCC^®^ HTB-38) human colorectal adenocarcinoma cells were cultured and maintained in McCoy’s 5A media supplemented with 10 % fetal bovine serum and 1 % penicillin-streptomycin. The culture was maintained at 37 °C, 5 % CO_2_ over air.

### 2.6. Cell metabolic activity in the dark

HT-29 cells (6 × 10^4^ cells) were seeded into a 96-well dish and grown to 80 % confluency. The media was replaced with different concentrations of added Rose Bengal Diacetate solution (RBD, 0 – 200 μM) in McCoy’s 5A media and the cells incubated in the dark for 24 h. The media was removed, and the cells incubated with a solution 3-(4,5-dimethylthiazol-2-yl)–2,5-diphenyltetrazolium bromide (MTT; 0.5 mg/mL) in water for 4 h. A solution of sodium dodecyl sulfate (0.1 g/mL) containing HCl (20 μM) was added to dissolve the crystals that had formed. The absorbance at 570 nm was recorded for each well and the experiments were conducted in triplicate for each RBD concentration. The average absorbance was determined as a measure of cell metabolic activity with the error bars corresponding to the standard deviation.

### 2.7. Photoinactivation of cell culture

For MTT activity assays, HT-29 cells (2.0 × 10^4^ cells) were seeded onto a 96-well plate and grown for 24 h. Rose Bengal Diacetate (30 μM) in McCoy’s 5A media was added, and the cells were incubated in the dark for 2.5 h. The surrounding media was removed, and the cells were placed in Minimum Essential Medium (MEM) media which lacks Phenol Red which is known to inhibit oxygen photosensitization efficiency [35, 38,39]. The wireless device was adhered to the outside bottom of the well containing the cells and the device was located approximately 1.5 cm from the transmitting antenna. The cells were irradiated for 0 – 40 min at room temperature, with 6 W of transmitting RF power and the laboratory lights off during the entire experiment to minimize ambient light interference. The MEM media was replaced with McCoy’s 5A media, and the cells allowed to recover for 16 h. Cell inactivation was evaluated utilizing the MTT activity assay, as described above and p values (probability of null hypothesis) were determined using an unpaired t-test from GraphPad. Data is an average of *N* = 3 for photoinactivation control experiments and an average of *N* = 5 experiments for the 0 – 40 min irradiation experiments.

For Lactase Dehydrogenase (LDH) cytotoxicity assays, HT-29 cells (0.5 × 10^4^ cells) were seeded onto a 96-well plate and grown for 24 h. Rose Bengal Diacetate (30 μM) in McCoy’s 5A media was added, and the cells were incubated in the dark for 2.5 h. The surrounding media was removed, and the cells were placed in MEM. The wireless device was adhered to the outside bottom of the well containing the cells and the device was located approximately 1.5 cm from the transmitting antenna. The cells were irradiated for 30 min at room temperature, with 6 W of transmitting RF power in the dark. LDH cytotoxicity assay (Milipore Sigma) was utilized to measure LDH release. Lysis buffer (5 μL) was added for 15 min in order to release maximum LDH as a positive control. To each well, 100 μL of LDH reaction buffer was added and incubated at 37 °C for 30 min. After the reaction stop solution (50 μL) was added, absorbance at 490 nm was recorded and average absorbance was used to measure cell death as the percentage of LDH released compared the positive control. P values were determined using an unpaired t-test from GraphPad. Data is an average of *N* = 3 LDH experiments.

### 2.8. Brightfield microscopic imaging after cell photoinactivation

HT-29 cells (2.0 × 10^4^) cells were seeded onto a 96-well dish and grown for 24 h. Rose Bengal Diacetate (30 μM) was added, and the cells were incubated in the dark for 2.5 h. The surrounding medium was removed and replaced with Minimum Essential Medium (MEM) which lacks Phenol Red. Before irradiation, brightfield microscopic images of the cells were acquired using a Keyence BZ-X810 microscope (20x). The device was adhered to the outside bottom of the well containing the cells and the device was located 0.5 cm from the transmitting antenna. The cells were irradiated for 30 min at room temperature. Brightfield microscopic images were captured immediately after irradiation and again 16 h post-irradiation.

### 2.9. Fluorescence microscopic imaging after cell photoinactivation

HT-29 cells (1.5 × 10^5^) cells were seeded onto a 35 mm glass bottom dish and grown for 24 h. Rose Bengal Diacetate (30 μM) was added, and the cells were incubated in the dark for 2.5 h. The surrounding medium was removed and replaced with Minimum Essential Medium (MEM) which lacks Phenol Red. The device was adhered to the bottom of the dish containing the cells. The cells were irradiated for 30 min at room temperature and incubated in the dark for 16 h. The MEM was removed and 1X Binding Buffer containing Annexin-V-FITC (5 μL of stock solution per 1 mL Binding Buffer) and Propidium Iodide (PI, 5 μL of stock solution per 1 mL Binding Buffer) was added to the cells with incubation for 5 min. The Binding Buffer was removed, and the cells washed with 1X PBS to remove any unbound fluorescent probe. MEM was added for cell fluorescence microscopy and images were captured 2 h post-irradiation and again at 16 h post-irradiation using a Zeiss Axiovert 100 TV epifluorescence microscope (10x) equipped with an X-Cite 120Q light source with metal-halide lamp. The images were acquired using FITC (Ex: 485/20 nm, Em: 524/24 nm) and TxRed (Ex: 562/40 nm, Em: 624/40 nm) filter sets.

## 3. Results

### 3.1. Device optical power evaluation

**Fig. 2.**
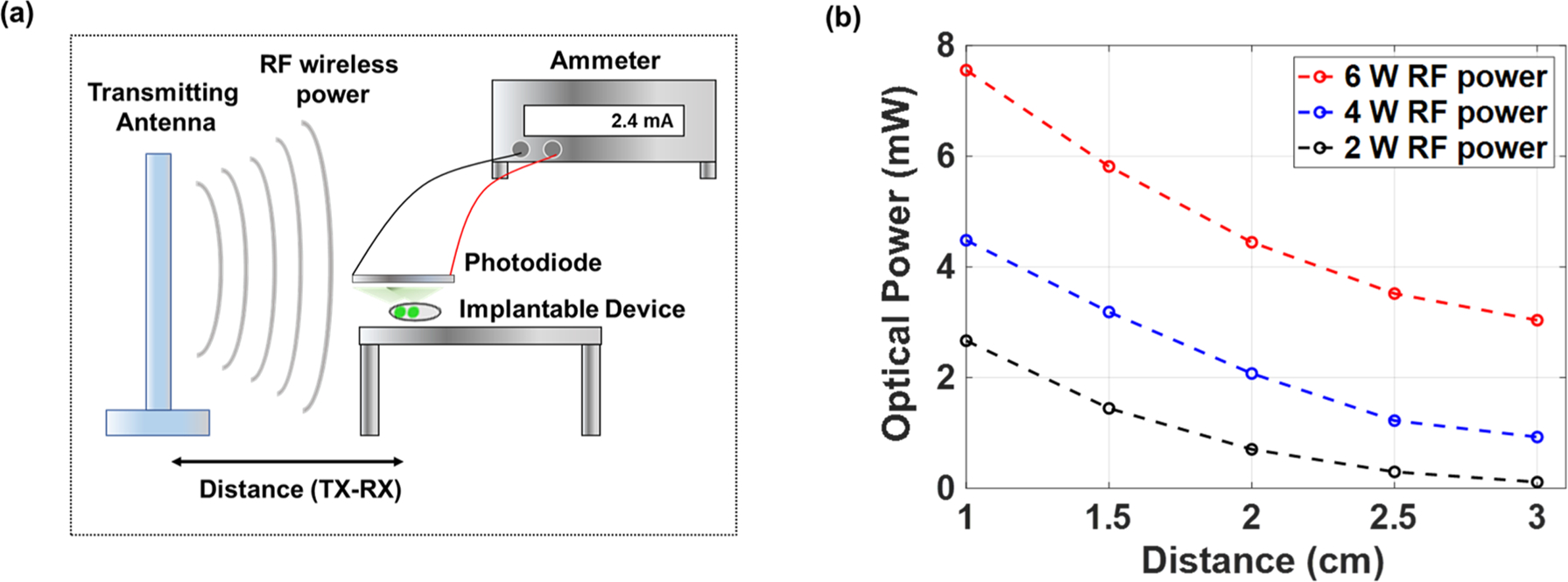
(a) Method used to measure optical output power of wireless optical device. (b) Measured optical output from device as a function of distance (TX-RX) from the transmitting antenna and antenna RF power.

The optical power output from the device was measured by placing a silicon photodiode directly on top of the LED (Fig. 2a). The radiant power was evaluated in relation to the distance between the device and transmitting antenna while employing multiple transmitting antenna power levels (2, 4, or 6 W). The plots in Fig. 2b indicate a maximum emitted optical output of nearly 8 mW when the separation distance was 1 cm. For each power setting, the measured optical output power presented a non-linear relationship with the separation distance. It is noted that LEDs are nonlinear devices: their impedance depends on operating voltage and their output power varies nonlinearly with voltage. Since the wireless power transfer efficiency depends on the impedance matching and transmitter distance, nonlinear radiant power is observed [40,41].

The dependence of radiant power on device orientation relative to the transmitting antenna was evaluated with systematic alteration of the orientation angles along the x, y, and z-axes. As illustrated in Figs. S1 and S2, the light output intensity varied little with device orientation angles of 0, 30, and 60° in the x, y, and z-axes. Only when the device orientation angle was close to 90° was there a significant drop in light intensity. Since the device directs its output light as a beam in a specific direction, we also evaluated the effects of rotating the device in tissue to examine different placement conditions. The photographs in Fig. S3 compare the light output for two devices buried in chicken breast with the beams directed in opposite directions (towards or away from the viewer). Due to multiple scattering in the tissue, significant light intensity is still observed through the tissue even when the LED is facing away from the viewer. Lastly, we assessed the attenuation of RF power when chicken breast was placed between the device and the antenna. As illustrated in Figs. S4 and S5, the presence of intervening chicken breast hardly altered the device light output intensity. Furthermore, the presence of intervening chicken breast did not alter the dependence of radiant power with distance between device and antenna (Fig. S6).

### 3.2. Photosensitization efficiency

**Fig. 3.**
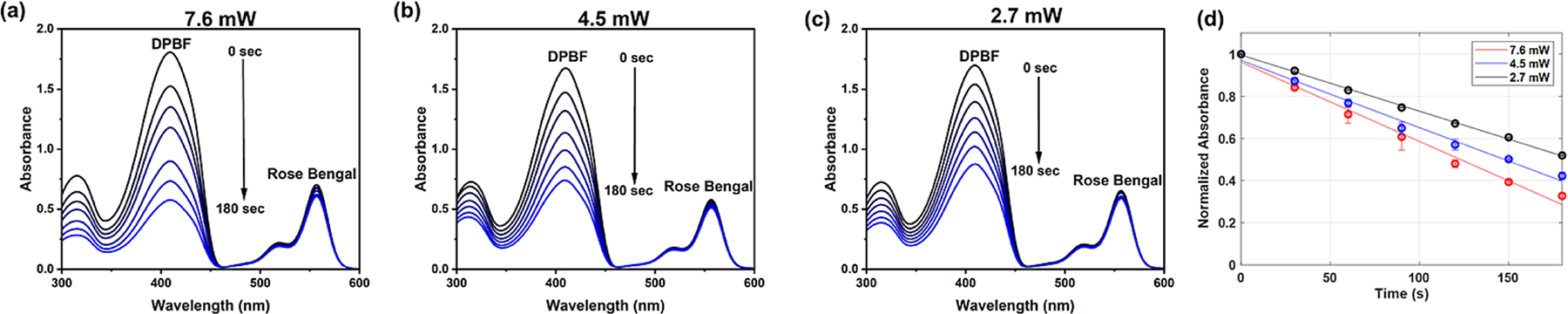
Measurements of singlet oxygen photogeneration. (a – c) Absorbance spectra of separate solutions containing 1,3-diphenylisobenzofuran (DPBF, 75 μM) and Rose Bengal (6 μM) in methanol and irradiated for 180 s with the wireless device at optical power of 7.6 mW, 4.5 mW, or 2.7 mW, respectively. (d) Plots of normalized DPBF absorption at 415 nm (*N* = 2) indicating the rate of photogenerated singlet oxygen produced by device irradiation. Error bars indicate estimated error of ±5 %.

The ability of the wireless device to photogenerate singlet oxygen was assessed by conducting a benchtop experiment using the chemical trap, 1,3-diphenylisobenzofuran (DPBF) [42]. DPBF absorbs light at 415 nm and reacts rapidly with singlet oxygen to generate a colorless endoperoxide product (Scheme S1). The decrease in DPBF absorbance reflects the rate of singlet oxygen photogeneration within an irradiated methanol solution that contained Rose Bengal as the PS. Production of singlet oxygen due to illumination from the device at multiple power levels (2, 4, or 6 W) was indicated by the decrease in absorbance at 415 nm (Fig. 3a–c). The rate of singlet oxygen production was reduced with lower transmitter power levels (Fig. 3d). These cuvette experiments demonstrate that the device can photogenerate moderate amounts singlet oxygen due to type II photosensitization by Rose Bengal.

### 3.3. Photodynamic cell inactivation studies

HT-29 (human colorectal adenocarcinoma) cells are commonly used for evaluation of photoinactivation strategies [43–46], and cultured monolayers of HT-29 cells were employed to assess photoinactivation efficiency using the wireless device in combination with Rose Bengal as the PS. The HT-29 cell cultures were treated with Rose Bengal Diacetate, a lipophilic derivative that is membrane permeable and quickly converted to Rose Bengal by intracellular esterases [8,36]. Interestingly, high concentrations of Rose Bengal Diacetate (> 100 μM) have been shown to induce cell death of colorectal cancer cells in the dark [47]. Therefore, it was important to ensure that the Rose Bengal Diacetate doses used in this study did not induce dark-state toxicity. Standard MTT cell metabolic activity assays were conducted that incubated HT-29 cells with increasing doses of Rose Bengal Diacetate in the dark for 24 h. As shown by the bar graph in Fig. S7, there was no loss in cell metabolic activity up to the highest concentration tested (200 μM). Another control experiment used the MTT assay to confirm that HT-29 cell metabolic activity was unchanged by RF (6 W) exposure for 40 min in the dark Fig. S8.

**Fig. 4.**
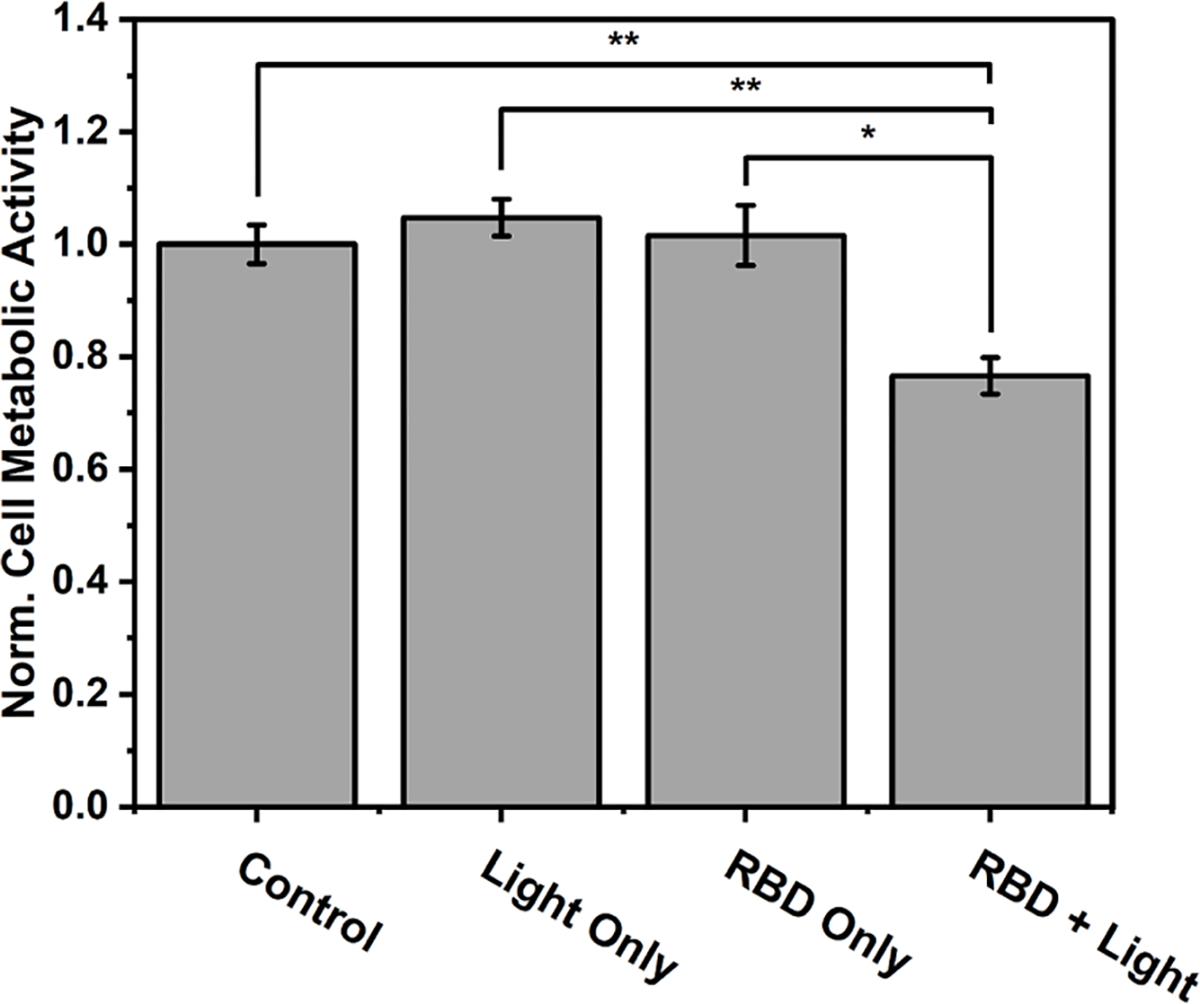
Normalized metabolic activity of HT-29 cell cultures measured by MTT assay. Cell treatment conditions: No treatment and room temperature as the control condition, light only (573 nm wireless device for 40 min), 30 μM Rose Bengal Diacetate (RBD) only, or 30 μM RBD + Light. Data is average of *N* = 3 experiments with error bars indicating standard deviation. * *p* < 0.05, ** *p* < 0.01.

**Fig. 5.**
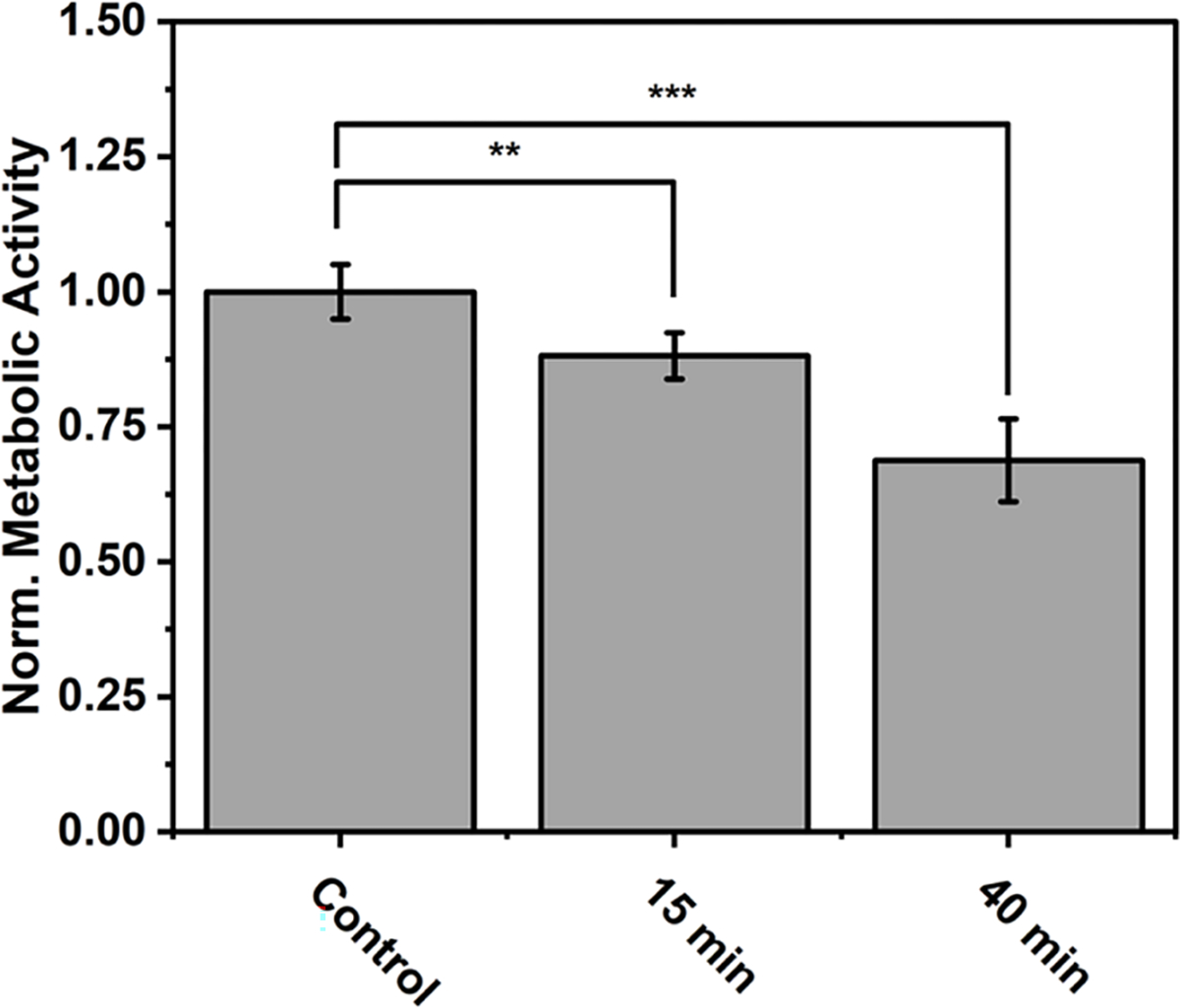
Normalized metabolic activity of HT-29 cell cultures measured by MTT assay. No treatment is the control condition. The other two conditions are 30 μM Rose Bengal Diacetate (RBD) + light (573 nm wireless device) for 15 min) or 40 min. Data is average of *N* = 5 experiments with error bars indicating standard deviation. *** *p* = 0.0003, ** *p* = 0.0065.

The cell photoinactivation studies subjected equivalent batches of HT-29 cell culture to the following different conditions: (a) untreated control, (b) Rose Bengal Diacetate treatment but no light, (c) only light exposure from the device, and (d) Rose Bengal Diacetate treatment plus light exposure from the device. The experimental workflow is outlined in Scheme S2. In short, the cells were cultured in a multiwell plate, and the device was adhered to the outside bottom of the well containing the cells. The cells were incubated with Rose Bengal Diacetate (30 μm) for 2.5 h before device irradiation for 40 min with 6 W of transmitting RF power. After a 16 h recovery period, cell metabolic activity was measured using the MTT assay. As indicated in Fig. 4, reduced cell metabolic activity was only observed in wells exposed to Rose Bengal Diacetate and light. A subsequent set of experiments evaluated the impact of light exposure time and found that a 40 min light exposure increased the photoinactivation effect compared to 15 min light exposure (Fig. 5). This data indicates that an extended light exposure time can progressively trigger more cell death. Additional photoinactivation studies quantified release of lactose dehydrogenase (LDH) which is diagnostic of membrane damage due to cell death and recorded about 20 % cell death (Fig. S7b) in agreement with the MTT data.

**Fig. 6.**
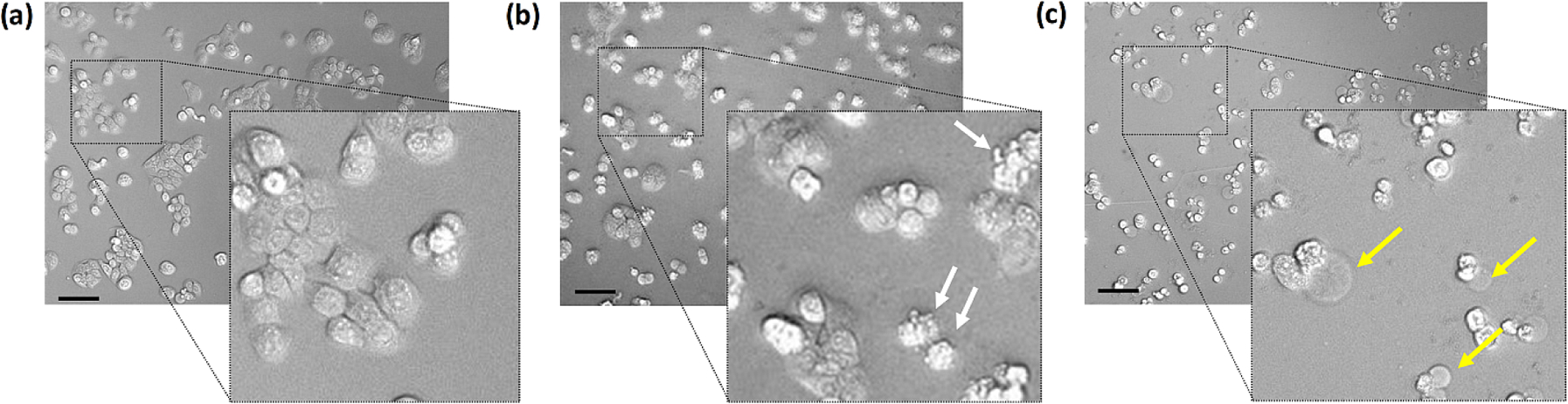
Brightfield microscopy images of HT-29 cells (20x). (a) Control cells, (b) Cells treated with Rose Bengal Diacetate (30 μM) and irradiated using the wireless device (30 min), imaged directly after irradiation, and (c) Cells imaged 16 h after irradiation. White arrows indicate membrane ruffling. Yellow arrows indicate protruding bubbles (pyroptotic bodies). Scale bar = 50 μm.

After confirming that the device can induce photoinactivation of HT-29 cells that had been treated with Rose Bengal Diacetate, we used brightfield microscopy to image the cell morphological changes induced by the photodynamic treatment. As illustrated in Figs. 6, and S9, a cultured monolayer of healthy HT-29 cells included cells that were either flattened and adherNIHMS1995703-supplement-MMC1.docxed, or they were stacked as a small group. Directly following a 30 min irradiation, brightfield microscopy revealed cell membrane ruffling with small extrusions, a common feature of dying cells [48]. Additional microscopy at 16 h post-irradiation showed many cells with relatively large bubble-like protrusions, a hallmark feature of pyroptosis, a cell death process that induces osmotic swelling of the plasma membrane [49,50]. In addition, phase contrast microscopy of the HT-29 cells confirmed the presence of the bubble-like protrusions (Fig. S10). Finally, fluorescence microscopy subcellular localization studies with HT-29 cells and RBD were performed and indicated localization of RBD to mitochondria (Fig. S11) which is consistent with previous studies suggesting that mitochondrial damage initiates pyroptosis [8].

**Fig. 7.**
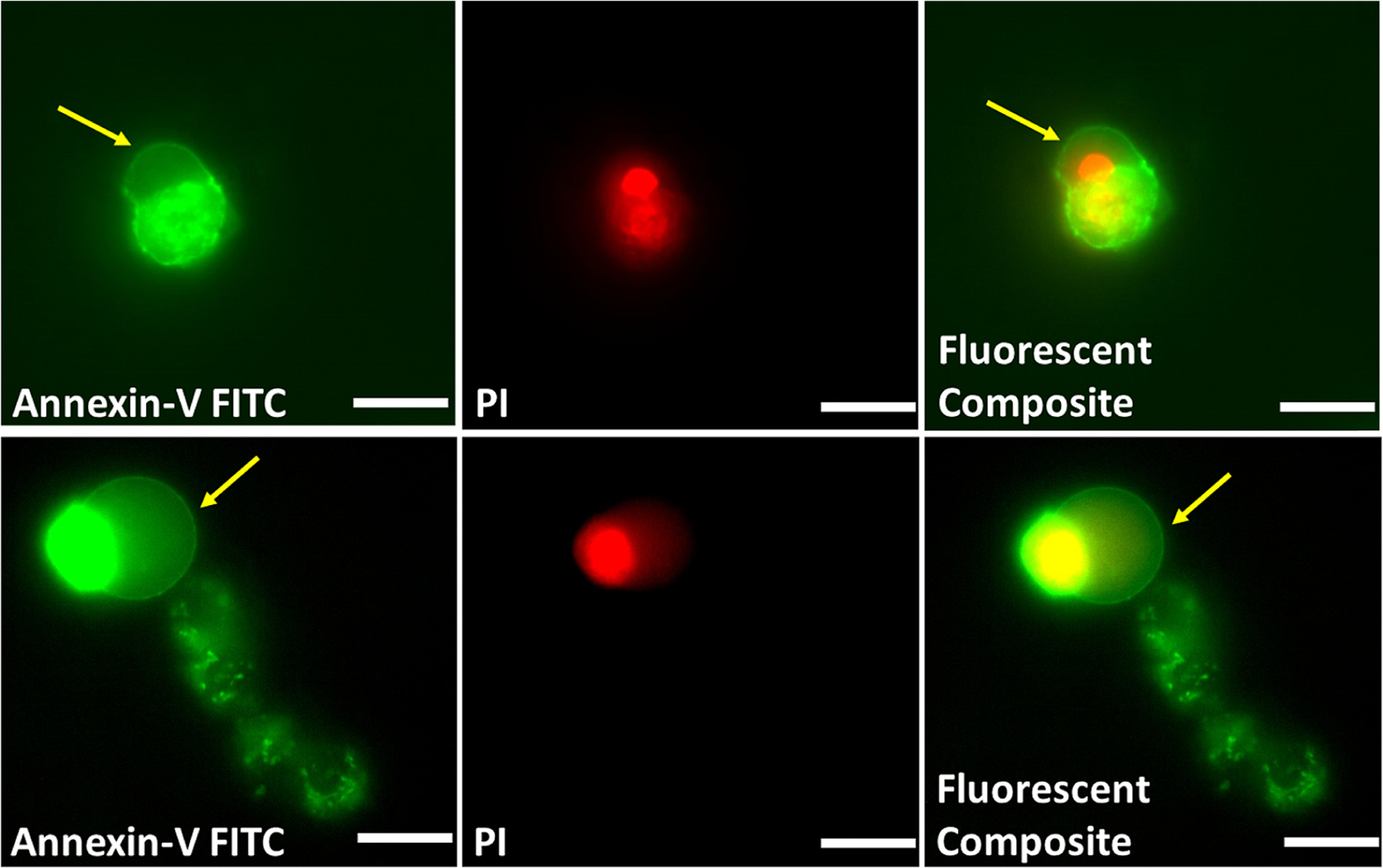
Two representative sets of dual-channel fluorescence microscopy images. Micrographs showing HT-29 cells treated with Rose Bengal Diacetate (30 μM), irradiated using the wireless device (30 min), allowed to incubate for 16 h, then stained with Annexin-V FITC (Ex: 485/20 nm, Em: 524/24 nm) and Propidium Iodide (PI) (Ex: 562/40, Em 624/40). Yellow arrow indicates a protruding bubble (pyroptotic body) in each case. Scale bars = 30 μm.

Supporting evidence for light-induced cell pyroptosis was gained by acquiring fluorescence micrographs of the cells treated with green emitting Annexin-V FITC and red emitting Propidium Iodide (PI). At 2 h post-irradiation there was prominent Annexin-V FITC staining of the cell membranes indicating exposed phosphatidylserine a signature of dead/dying cells, but very few protruding bubbles (Fig. S12). At 16 h post-irradiation there was noticeable increase in the number of protruding bubbles and very strong PI staining of the cytosol indicating a permeable membrane (Fig. 7). This staining pattern is consistent with pyroptosis as a contributing cell death pathway and suggests that apoptosis cannot be the only cell death pathway because apoptosis is a process that retains an impermeable plasma membrane and prevents cell entry of PI. A final cell microscopy experiment exploited the large changes in cell morphology to determine the size of the light-induced cell killing zone around the miniature device. The brightfield micrograph in Fig. S13 shows a circular petri dish of HT-29 cells that had been treated with Rose Bengal Diacetate (30 μM) and irradiated with a miniature device located in the middle of the plate. The image was captured 16 h post irradiation and reveals a distinctive crossover from healthy cell morphology to dead cell morphology at a zone radius of about 4 mm from the device. Undoubtedly, this cell killing radius would be further reduced in a therapeutic circumstance that surrounds the device with biological tissue, but as discussed below complete light-induced tumor cell death is not envisioned as the therapeutic goal. The decrease in photoinactivation with distance explains why the above cell death assays (MTT and LDH release) only showed only 20 % photoinactivation of the cells within an irradiated microwell because the distant cells received less light intensity from the miniature device. In other words, light irradiation was not uniform over the entire microwell.

## 4. Discussion

A longstanding limitation of phototherapy is the challenge of delivering sufficient light power to deep-tissue sites of disease such as cancerous tumors. A wireless implantable light source is an attractive strategy for light delivery that can provide potential benefits for preclinical research and perhaps eventually clinical treatment. In terms of preclinical research, there are emerging treatment concepts under investigation such as metronomic PDT which involves intermittent bursts of low power irradiation over an extended period (up to several days) [19]. Small animal research on this method would be greatly facilitated if the light could be delivered by an implanted LED that only had to be inserted once at the beginning of the extended treatment and could be powered by a programable remote antenna outside the housing cage. Clinically, an implanted LED may represent a path toward realizing metronomic PDT without requiring multiple surgical or endoscopy procedures.

Wireless powering of medical implants such as a miniature LED can be achieved by different methods and each has distinctive features [51]. For example, solar power is attractive for long term applications [52] and ultrasonic wireless power transfer is well-suited for deeper and smaller implants, albeit with less power efficiency and more complexity [21]. Inductive coupling is more commonly employed due its higher efficiency and commercial availability of electronic components [20, 22–29]. The miniature wireless LED-based device described here is one of the smallest RF inductively powered prototypes that has been tested to date and is small enough to be implanted into a living subject using a standard biopsy syringe. The device design includes impedance matching of two LEDs that are secured in an antiparallel configuration to a miniature helical antenna. The wireless transfer of RF power is effective even when the device is buried in chicken breast and located 6 cm from the transmitting antenna (Fig. S6).

Current LED technology allows the light output wavelength to be customized to a desired value. While longer wavelengths (> 660 nm) penetrate more deeply through biological tissue, we deliberately chose LEDs that produced green light (573 nm) because it closely matches the excitation wavelength of Rose Bengal which is a very efficient oxygen PS that can kill cancer and microbial cells by PDT [8,31–34]. The capacity of Rose Bengal to produce phototoxic effects with low intensity light makes it a good choice as the PS for excitation by a miniature wireless LED-based device with limited radiant power. Moreover, Rose Bengal is an FDA-approved vital dye that has been used clinically for more than three decades in diagnostic ophthalmology procedures [31]. PDT using Rose Bengal has been investigated extensively for multiple potential therapeutic applications and there is evidence that it can trigger cell death process that are immunogenic, thus suggesting a future way to treat disease at tissue locations that are distant to the specific site of irradiation [8,35–53]. A 2011 review of PDT using cell permeable Rose Bengal Diacetate summarized evidence for apoptosis, autophagy, and necrosis as independent cell death pathways [36]. This current study observed cell morphology changes, LDH release, and fluorescence staining patterns that indicate light-induced pyroptosis as a significant cell death mechanism. To the best of our knowledge this is the first time that light induced pyroptosis has been implicated as a contributing cell death pathway for photoinactivation using Rose Bengal Diacetate as the PS. Twenty-year old PDT studies, using HT-29 colorectal adenocarcinoma cells and PS dyes with deep-red excitation wavelengths, concluded that low-dose light induces apoptosis whereas high-dose light tends to cause necrosis [45]. It is not clear if the switch to green wavelength Rose Bengal Diacetate as the PS is the reason why pyroptosis is induced or if the previous studies were simply unaware of pyroptosis since it is a newly discovered cell death pathway [13,54].

The results suggest that our wireless implanted LED-based device has long-term potential as a new way to induce phototherapy of deep-seated cancerous tissue. However, it is presently not clear if an implantable wireless LED will offer any clinical improvement over thin optical fibers which can also be introduced into deep tissue locations. Additional study is necessary to directly compare the advantages and disadvantages of each technique, and whether continuous “untethered” treatment outside of a clinical setting is safe. For example, injection of an implanted LED may result in local bleeding that can impede light transmission and lessen treatment effects. Further *in vitro* and *in vivo* studies are necessary to resolve these issues before clinical translation. One goal of future work is to determine the scope of cell-lines that can be induced to undergo light-induced pyroptosis using Rose Bengal PDT, and the extent of pyroptosis relative to other cell death pathways. Apoptosis is commonly considered a non-immunogenic process and diversion of the cell death process towards immunogenic pyroptosis might be an effective way to generate a strong immune response in a living subject [13]. Using light to do this raises the interesting idea of sensing feedback and real-time control, since unchecked levels of pyroptosis can promote strong inflammatory reactions that can lead ultimately to organ damage or septic shock [55]. As a free dye, Rose Bengal does not accumulate in malignant cells specifically but there are ways to package it within tumor targeting nanoparticles [32]. While the limited penetration of green light through tissue restricts the irradiation volume, it is worth emphasizing that the envisioned therapeutic goal is not necessarily to kill directly all the tumor cells but rather to convert an immune-silent tumor into an immunogenic tumor by stimulating the activation and recruitment of immune cells. We note that a recent study using a mouse mammary tumor graft found that pyroptosis of less than 15 % of the tumor cells was sufficient to eradicate the entire tumor [56]. Regardless, the implantable LED can be readily adapted for use with a longer-wavelength PS, that will allow deeper of the light penetration through tissue.

## 5. Conclusion

This study developed a miniature LED-based device with a volume of 23 mm^3^ which is one of the smallest prototypes to date. The device is powered wirelessly by RF energy and emits light with a wavelength of 573 nm which can excite Rose Bengal as an efficient oxygen PS for potential use in PDT. The wireless transfer of RF power is effective even when the device is buried in chicken breast and located 6 cm from the transmitting antenna. Studies of cell cultures comprised of HT-29 colorectal adenocarcinoma cells treated with cell permeable Rose Bengal Diacetate observed cell photoinactivation by pyroptosis as judged by a diagnostic combination of cell morphology changes and staining by fluorescent probes. A goal of future PDT work is to determine if light-induced pyroptosis using Rose Bengal Diacetate is possible in living subjects. If the use of 573 nm irradiation is not practical due to limited penetration of the light through biological tissue, we can readily switch to a complementary LED/PS pair that is based on longer wavelength light. An attractive idea is to deploy energy up-conversion nanoparticles that enable excitation of encapsulated Rose Bengal using long wavelength light [18]. Additional long term directions are, (a) make smaller LEDs which can expand the possible therapeutic applications but will likely diminish light power, and (b) combine the LED emission with sensing and drug release technologies to create multifunctional devices with feedback capabilities [57]. We are especially interested in a wireless implanted LED-based device that can trigger and self-report activation of an immunogenic cell death process like pyroptosis in deep-seated cancerous tissue [58]. We envision new methods for neoadjuvant therapy that use the implanted device to trigger an immune response that can attack distant cancer sites. A related concept is photodynamic priming which sensitizes tumors to secondary therapies, such as immunotherapy [59]. It is worth highlighting that Rose Bengal can be used to treat certain cancers in the absence of light [47,60]. which raises the alluring possibility that Rose Bengal treatments can be designed to kill cancer cells by a complementary combination of dark and light-based methods. Furthermore, photoexcited Rose Bengal can activate other photochemical reactions that can become the basis of entirely new classes of phototherapies [61]. A final point is that green light has enough energy to cleave chemical bonds and thus activate drug release and biological uncaging or optogenetic processes that can become the basis of new methods in biomedical research and clinical treatment [11,12,14].

## Supplementary Material

MMC1

## References

[1] Algorri JF, Ochoa M, Roldán-Varona P, Rodríguez-Cobo L, López-Higuera JM, Light technology for efficient and effective photodynamic therapy: a critical review, Cancers 13 (2021) 3484, 10.3390/cancers13143484. 34298707 PMC8307713

[2] Kim HJ, Sritandi W, Xiong Z, Ho JS, Bioelectronic devices for light-based diagnostics and therapies, Biophys. Rev. 4 (2023) 11304, 10.1063/5.0102811. PMC10903427 38505817

[3] Yun SH, Kwok SJJ, Light in diagnosis, therapy and surgery, Nat. Biomed. Eng. 1 (2017) 0008, 10.1038/s41551-016-0008. 28649464 PMC5476943

[4] Kessel D, Photodynamic therapy: a brief history, J. Clin. Med. 8 (2019) 1581, 10.3390/jcm8101581. 31581613 PMC6832404

[5] Sobhani N, Samadani AA, Implications of photodynamic cancer therapy: an overview of PDT mechanisms basically and practically, J. Egypt Natl. Cancer Inst. 33 (2021) 1–13, 10.1186/s43046-021-00093-1. PMC13316895 34778919

[6] Deng K, Li C, Huang S, Xing B, Jin D, Zeng Q, Hou Z, Lin J, Recent progress in near infrared light triggered photodynamic therapy, Small 13 (2017) 1702299, 10.1002/smll.201702299. 28961374

[7] Fernandez JM, Bilgin MD, Grossweiner LI, Singlet oxygen generation by photodynamic agents, J. Photochem. Photobiol. B 37 (1997) 13–140.

[8] Panzarini E, Inguscio V, Dini L, Timing the multiple cell death pathways initiated by rose bengal acetate photodynamic therapy, Cell Death Dis. 2 (2011) e169, 10.1038/cddis.2011.51. 21654827 PMC3168993

[9] Mishchenko T, Balalaeva I, Gorokhova A, Vedunova M, Krysko DV, Which cell death modality wins the contest for photodynamic therapy of cancer? Cell Death Dis. 13 (2022) 1–16, 10.1038/s41419-022-04851-4. PMC9106666 35562364

[10] Zhao X, He S, Wang J, Ding J, Zong S, Li G, Sun W, Du J, Fan J, Peng X, Near-infrared self-assembled hydroxyl radical generator based on photoinduced cascade electron transfer for hypoxic tumor phototherapy, Adv. Mater. 35 (2023) e2305163, 10.1002/adma.202305163. 37545041

[11] Weinstain R, Slanina T, Kand D, Klán P, Visible-to-NIR-light activated release: from small molecules to nanomaterials, Chem. Rev. 120 (2020) 13135–13272, 10.1021/acs.chemrev.0c00663. 33125209 PMC7833475

[12] Ryu KA, Kaszuba CM, Bissonnette NB, Oslund RC, Fadeyi OO, Interrogating biological systems using visible-light-powered catalysis, Nat. Rev. Chem. 5 (2021) 322–337, 10.1038/s41570-021-00265-6. 37117838

[13] Li M, Kim J, Rha H, Son S, Levine MS, Xu Y, Sessler JL, Kim JS, Photon-controlled pyroptosis activation (PhotoPyro): an emerging trigger for antitumor immune response, J. Am. Chem. Soc. 145 (2023) 6007–6023, 10.1021/jacs.3c01231. 36881923 PMC12962054

[14] Bonnet S, Why develop photoactivated chemotherapy? Dalton Trans. 47 (2018) 10330–10343, 10.1039/c8dt01585f. 29978870

[15] Choi SK, Photoactivation strategies for therapeutic release in nanodelivery systems, Adv. Ther. 3 (2020) 2000117, 10.1002/adtp.202000117.

[16] Sun B, Bte Rahmat JN, Zhang Y, Advanced techniques for performing photodynamic therapy in deep-seated tissues, Biomater 291 (2022) 121875, 10.1016/j.biomaterials.2022.121875. 36335717

[17] Zhang W, Zhang X, Shen Y, Shi F, Song C, Liu T, Gao P, Lan B, Liu M, Wang S, Fan L, Lu H, Ultra-high FRET efficiency NaGdF4: tb3+-rose bengal biocompatible nanocomposite for X-ray excited photodynamic therapy application, Biomater 184 (2018) 31–40, 10.1016/j.biomaterials.2018.09.001. 30195803

[18] Nahorniak M, Pop-Georgievski O, Velychkivska N, Filipová M, Rydvalová E, Gunár K, Matouš P, Kostiv U, Horák D, Rose bengal-modified upconverting nanoparticles: synthesis, characterization, and biological evaluation, Life 12 (2022) 1383, 10.3390/life12091383. 36143419 PMC9502678

[19] Lin T, Zou P, Lin R, Guan H, Fang Z, Chen J, Long Z, Zhang Y, Xing L, Qi F, Lang J, Xue X, Chen M, A self-powered wireless detachable drug/light injector for metronomic photodynamic therapy in cancer treatment, Nano Energy 116 (2023) 108826, 10.1016/j.nanoen.2023.108826.

[20] Jeong SH, Lee MG, Kim CC, Park J, Baek Y, Park BI, Doh J, Sun JY, An implantable ionic therapeutic platform for photodynamic therapy with wireless capacitive power transfer, Mater. Horiz. 10 (2023) 2215–2225, 10.1039/d2mh01548j. 37000519

[21] Kim A, Zhou J, Samaddar S, Song SH, Elzey BD, Thompson DH, Ziaie B, An implantable ultrasonically-powered micro-light-source (μLight) for photodynamic therapy, Sci. Rep. 9 (2019) 1–9, 10.1038/s41598-019-38554-2. 30718792 PMC6362227

[22] Choi J, Lee IS, Lee JS, Jeon S, Yun WS, Yang S, Moon Y, Kim J, Kim J, Choy S, Jeong C, Shim MK, il Kim T, Kim K, Implantable micro-scale LED device guided photodynamic therapy to potentiate antitumor immunity with mild visible light, Biomater. Res. 26 (2022) 1–15, 10.1186/s40824-022-00305-2. 36258234 PMC9580183

[23] Bansal A, Yang F, Xi T, Zhang Y, Ho JS, *In vivo* wireless photonic photodynamic therapy, Proc. Natl. Acad. Sci. U. S. A. 115 (2018) 1469–1474, 10.1073/pnas.1717552115. 29378941 PMC5816188

[24] Kirino I, Fujita K, Sakanoue K, Sugita R, Yamagishi K, Takeoka S, Fujie T, Uemoto S, Morimoto Y, Metronomic photodynamic therapy using an implantable LED device and orally administered 5-aminolevulinic acid, Sci. Rep. 10 (2020) 1–11, 10.1038/s41598-020-79067-7. 33328544 PMC7744509

[25] Yamagishi K, Kirino I, Takahashi I, Amano H, Takeoka S, Morimoto Y, Fujie T, Tissue-adhesive wirelessly powered optoelectronic device for metronomic photodynamic cancer therapy, Nat. Biomed. Eng. 3 (2019) 27–36, 10.1038/s41551-018-0261-7. 30932063

[26] Sun B, Bte Rahmat JN, Kim HJ, Mahendran R, Esuvaranathan K, Chiong E, Ho JS, Neoh KG, Zhang Y, Wirelessly activated nanotherapeutics for in vivo programmable photodynamic-chemotherapy of orthotopic bladder cancer, Adv. Sci. 9 (2022) 2200731, 10.1002/advs.202200731. PMC9165499 35393785

[27] Yokoi K, Yasuda Y, Kanbe A, Imura T, Aoki S, Development of wireless power-transmission-based photodynamic therapy for the induction of cell death in cancer cells by cyclometalated iridium(III) complexes, Molecules 28 (2023) 1433, 10.3390/molecules28031433. 36771099 PMC9919167

[28] Kim K, Min IS, Kim TH, Kim DH, Hwang S, Kang K, Kim K, Park S, Lee J, Cho YU, Lee JW, Yeo WH, Song YM, Jung Y, Yu KJ, Fully implantable and battery-free wireless optoelectronic system for modulable cancer therapy and real-time monitoring, NPJ Flex 7 (2023) 41, 10.1038/s41528-023-00276-x.

[29] Nakajima K, Kimura T, Takakura H, Yoshikawa Y, Kameda A, Shindo T, Sato K, Kobayashi H, Ogawa M, Implantable wireless powered light emitting diode (LED) for near-infrared photoimmunotherapy: device development and experimental assessment in vitro and in vivo, Oncotarget 9 (2004) 20048–20057. www.oncotarget.com. 10.18632/oncotarget.25068 PMC5929445 29732002

[30] De Almeida AJPO, De Oliveira JCPL, Da Silva Pontes LV, De Souza Júnior JF, Gonçalves TAF, Dantas SH, De Almeida Feitosa MS, Silva AO, De Medeiros IA, ROS: basic concepts, sources, cellular signaling, and its implications in aging pathways, Oxid. Med. Cell Longev. 2022 (2022) 1225578, 10.1155/2022/1225578. 36312897 PMC9605829

[31] Vanerio N, Stijnen M, De Mol BAJM, Kock LM, Biomedical applications of photo- and sono-activated rose bengal: a review, Photobiomodul. Photomed. Laser Surg. 37 (2019) 383–394, 10.1089/photob.2018.4604. 31180251

[32] Demartis S, Obinu A, Gavini E, Giunchedi P, Rassu G, Nanotechnology-based rose bengal: a broad-spectrum biomedical tool, Dyes Pigments 188 (2021) 109236, 10.1016/j.dyepig.2021.109236.

[33] Pierański MK, Kosiński JG, Szymczak K, Sadowski P, Grinholc M, Antimicrobial photodynamic inactivation: an alternative for group b streptococcus vaginal colonization in a murine experimental model, Antioxid 12 (2023) 847, 10.3390/antiox12040847. PMC10135335 37107222

[34] Bartusik-Aebisher D, Ożóg Ł, Domka W, Aebisher D, Rose bengal and future directions in larynx tumor photodynamic therapy†, Photochem. Photobiol. 97 (2021) 1445–1452, 10.1111/php.13488. 34287926

[35] Panzarini E, Inguscio V, Fimia GM, Dini L, Rose bengal acetate photodynamic therapy (RBAc-PDT) induces exposure and release of damage-associated molecular patterns (DAMPs) in human HeLa cells, PLoS ONE 9 (2014) e105778, 10.1371/journal.pone.0105778. 25140900 PMC4139382

[36] Panzarini E, Inguscio V, Dini L, Overview of cell death mechanisms induced by rose bengal acetate-photodynamic therapy, Int. J. Photoenergy 2011 (2011) 713726, 10.1155/2011/713726.

[37] Rho S, Stillwell RA, Fay P, Ludwig KK, O’Sullivan TD, Optically-enhanced wireless breast lesion localization device for use during lumpectomy, in: Proceedings of the SPIE 119490F, 2022, p. 45, 10.1117/12.2606061.

[38] Gerdes R, Wöhrle D, Spiller W, Schneider G, Schnurpfeil G, Schulz-Ekloff G, Photo-oxidation of phenol and monochlorophenols in oxygen-saturated aqueous solutions by different photosensitizers, J. Photochem. Photobiol. A 111 (1997) 65–74, 10.1016/S1010-6030(97)00209-8.

[39] Head CS, Luu Q, Sercarz J, Saxton R, Photodynamic therapy and tumor imaging of hypericin-treated squamous cell carcinoma, World J. Surg. Oncol. 4 (2006) 87, 10.1186/1477-7819-4-87. 17147827 PMC1762016

[40] Alexeev A, Linnartz JPMG, Arulandu K, Deng X, Characterization of dynamic distortion in LED light output for optical wireless communications, Photonics Res. 9 (2021) 916, 10.1364/prj.416269.

[41] Elgala H, Mesleh R, Haas H, A study of LED nonlinearity effects on optical wireless transmission using OFDM, in: Proceedings of the IFIP International Conference on Wireless and Optical Communications Networks, 2009, pp. 1–5, 10.1109/WOCN.2009.5010576.

[42] Arunkumar E, Sudeep PK, Kamat PV, Noll BC, Smith BD, Singlet oxygen generation using iodinated squaraine and squaraine-rotaxane dyes, New J. Chem. 31 (2007) 677–683, 10.1039/b616224j. PMC2849118 20376333

[43] Nompumelelo Simelane NW, Kruger CA, Abrahamse H, Photodynamic diagnosis and photodynamic therapy of colorectal cancer: in vitro and in vivo, RSC Adv. 10 (2020) 41560–41576, 10.1039/d0ra08617g. 35516575 PMC9058000

[44] Wu J, Xiao Q, Zhang N, Xue C, Leung AW, Zhang H, Xu C, Tang QJ, Photodynamic action of palmatine hydrochloride on colon adenocarcinoma HT-29 cells, Photodiagnosis Photodyn. Ther. 15 (2016) 53–58, 10.1016/j.pdpdt.2016.05.005. 27181460

[45] Marchal S, Fadloun A, Maugain E, D’Hallewin MA, Guillemin F, Bezdetnaya L, Necrotic and apoptotic features of cell death in response to Foscan^®^ photosensitization of HT29 monolayer and multicell spheroids, Biochem. Pharmacol. 69 (2005) 1167–1176, 10.1016/j.bcp.2005.01.021. 15794937

[46] Bourré L, Simonneaux G, Ferrand Y, Thibaut S, Lajat Y, Patrice T, Synthesis, and *in vitro* and *in vivo* evaluation of a diphenylchlorin sensitizer for photodynamic therapy, J. Photochem. Photobiol. B 69 (2003) 179–192, 10.1016/S1011-1344(03)00020-4. 12695032

[47] Qin J, Kunda N, Qiao G, Calata JF, Pardiwala K, Prabhakar BS, Maker AV, Colon cancer cell treatment with rose bengal generates a protective immune response via immunogenic cell death, Cell Death Dis. 8 (2017) e2584, 10.1038/cddis.2016.473. 28151483 PMC5386459

[48] Battistelli M, Falcieri E, Apoptotic bodies: particular extracellular vesicles involved in intercellular communication, Biology 9 (2020) 21, 10.3390/biology9010021. 31968627 PMC7168913

[49] Wang S, Liu Y, Zhang L, Sun Z, Methods for monitoring cancer cell pyroptosis, Cancer Biol. Med. 19 (2022) 398–414, 10.20892/j.issn.2095-3941.2021.0504. PMC9088190 34931767

[50] de Vasconcelos NM, Van Opdenbosch N, Van Gorp H, Parthoens E, Lamkanfi M, Single-cell analysis of pyroptosis dynamics reveals conserved GSDMD-mediated subcellular events that precede plasma membrane rupture, Cell Death Differ. 26 (2019) 146–161, 10.1038/s41418-018-0106-7. 29666477 PMC6294780

[51] Haerinia M, Shadid R, Wireless power transfer approaches for medical implants: a review, Signals 1 (2020) 209–229, 10.3390/signals1020012.

[52] Kim J, Seo J, Jung D, Lee T, Ju H, Han J, Kim N, Jeong J, Cho S, Seol JH, Lee J, Active photonic wireless power transfer into live tissues, Proc. Natl. Acad. Sci. U. S. A. 117 (2020) 16856–16863, 10.1073/pnas.2002201117. 32632002 PMC7382277

[53] Panzarini E, Inguscio V, Tenuzzo BA, Dini L, *In vitro* and *in vivo* clearance of rose bengal acetate-photodynamic therapy-induced autophagic and apoptotic cells, Exp. Biol. Med. 238 (2013) 765–778, 10.1177/1535370213494552. 23828594

[54] Tan Y, Chen Q, Li X, Zeng Z, Xiong W, Li G, Li X, Yang J, Xiang B, Yi M, Pyroptosis: a new paradigm of cell death for fighting against cancer, J. Exp. Clin. Cancer Res. 40 (2021) 153, 10.1186/s13046-021-01959-x. 33941231 PMC8091792

[55] Chen M, Liao H, Bu Z, Wang D, Fang C, Liang X, Li H, Liu J, Zhang K, Su D, Pyroptosis activation by photodynamic-boosted nanocatalytic medicine favors malignancy recession, Chem. Eng. J. 441 (2022) 136030, 10.1016/j.cej.2022.136030.

[56] Wang Q, Wang Y, Ding J, Wang C, Zhou X, Gao W, Huang H, Shao F, Liu Z, A bioorthogonal system reveals antitumour immune function of pyroptosis, Nature 579 (2020) 421–426, 10.1038/s41586-020-2079-1. 32188939

[57] Zhang H, Peng Y, Zhang N, Yang J, Wang Y, Ding H, Emerging optoelectronic devices based on microscale LEDs and their use as implantable biomedical applications, Micromachines 13 (2022) 1069, 10.3390/mi13071069. 35888886 PMC9323269

[58] Alzeibak R, Mishchenko TA, Shilyagina NY, Balalaeva IV, Vedunova MV, Krysko DV, Targeting immunogenic cancer cell death by photodynamic therapy: past, present and future, J. Immunother. Cancer 9 (2021) e001926, 10.1136/jitc-2020-001926. 33431631 PMC7802670

[59] Bhandari C, Moffat A, Fakhry J, Malkoochi A, Nguyen A, Trinh B, Hoyt K, Story MD, Hasan T, Obaid G, A single photodynamic priming protocol augments delivery of ⍺-PD-L1 mAbs and induces immunogenic cell death in head and neck tumors, Photochem. Photobiol. (2023) 1–12, 10.1111/php.13865. PMC11006828 37818742

[60] Zhang L, Du J, Song Q, Zhang C, Wu X, A novel *in situ* dendritic cell vaccine triggered by rose bengal enhances adaptive antitumour immunity, J. Immunol. Res. 2022 (2022) 1178874, 10.1155/2022/1178874. 35155685 PMC8824725

[61] Ludvíková L, Friš P, Heger D, Šebej P, Wirz J, Klán P, Photochemistry of rose bengal in water and acetonitrile: a comprehensive kinetic analysis, Phys. Chem. Chem. Phys. 18 (2016) 16266–16273, 10.1039/C6CP01710J. 27253480

