# Supplementary material for: Miniature wireless LED-device for photodynamic-induced cell pyroptosis
Source: Photodiagnosis Photodyn Ther. Author manuscript; Available in PMC 2024 Aug 21. (PMC11336689; doi:10.1016/j.pdpdt.2024.104209)
Supplement: MMC1 [file NIHMS1995703-supplement-MMC1.docx]

**Supplementary Materials**

**Figure S1**. The xy-axis and xz-axis evaluation of the LEDs.

**Figure S2.** The measured radiant power in the different y-angles.

**Figure S3**. Diffusion of device output light due to passage through chicken breast.

**Figure S4**. Effect of chicken breast between device and antenna.

**Figure S5**. Optical output measurement without and with chicken breast.

**Figure S6**. Change in light output intensity with device-antenna distance in air vs device in chicken breast.

**Figure S7**. Normalized metabolic activity (MTT assay) of HT-29 cells and LDH cytotoxicity.

**Figure S8**. HT-29 cell metabolic activity (MTT assay) after RF exposure (6 W) at room temperature for 40 minutes in the dark.

**Figure S9**. HT-29 cell morphology changes caused by photoinactivation.

**Figure S10**. Phase contrast imaging of HT-29 morphological changes.

**Figure S11**. Subcellular localization of Rose Bengal Diacetate.

**Figure S12**. Fluorescence microscopy of photoinactivated HT-29 cells.

**Figure S13**. Distance dependence on cell photoinactivation.

**Scheme S1**. The reaction scheme of 1,3-diphenylisobenzofuran (DPBF) with singlet oxygen to produce a colorless product.

**Scheme S2**. Workflow of HT-29 cell photosensitization experiment with miniature LED-based device.

1. **
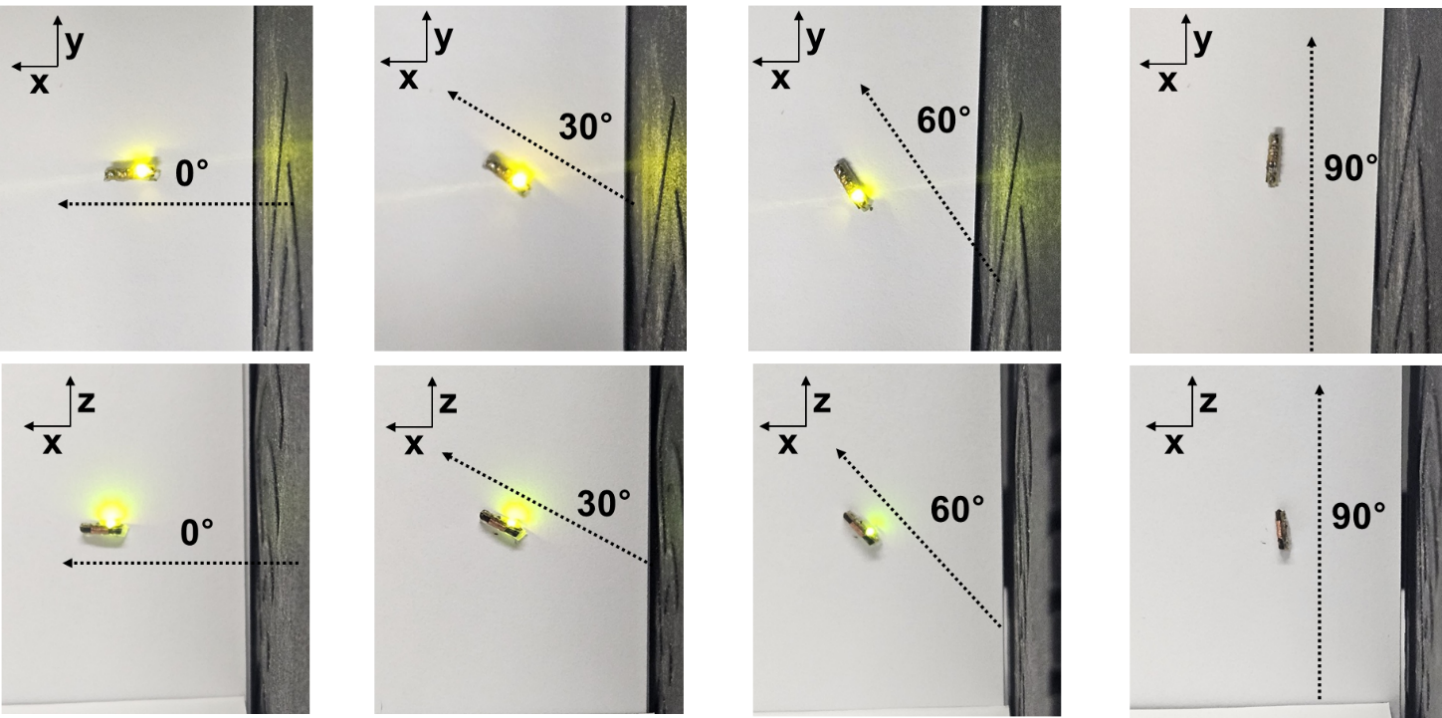
Evaluation of LED-based Device**

**Figure S1. The xy-axis and xz-axis evaluation of the LEDs.** The device is positioned at various angles along the y-axis and z-axis. (The orientation is determined based on the direction from the TX antenna to the LED of the device). The LED emits light at wide angles along the y-axis and z-axis.


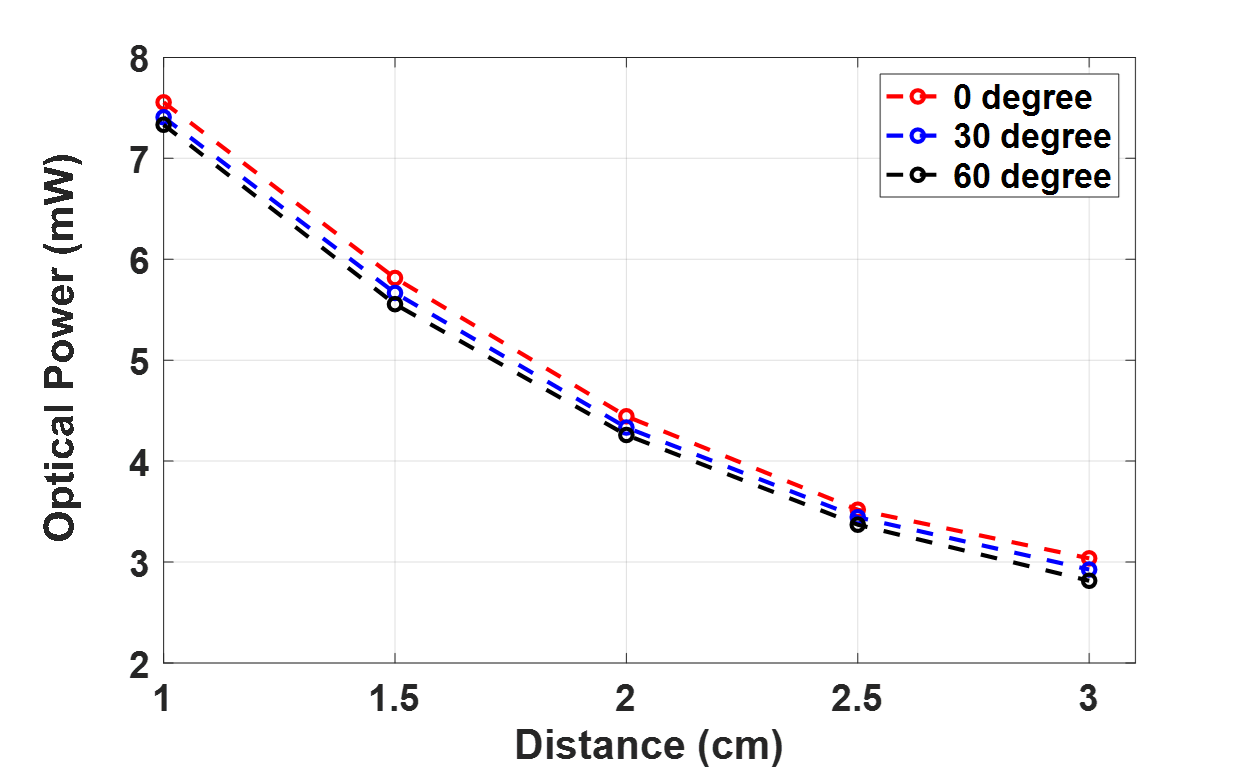


**Figure S2. The radiant power in the different y-angles.** The device is positioned at various angles along the y-axis at 6 W. The output power of the device is measured as a function of distance.


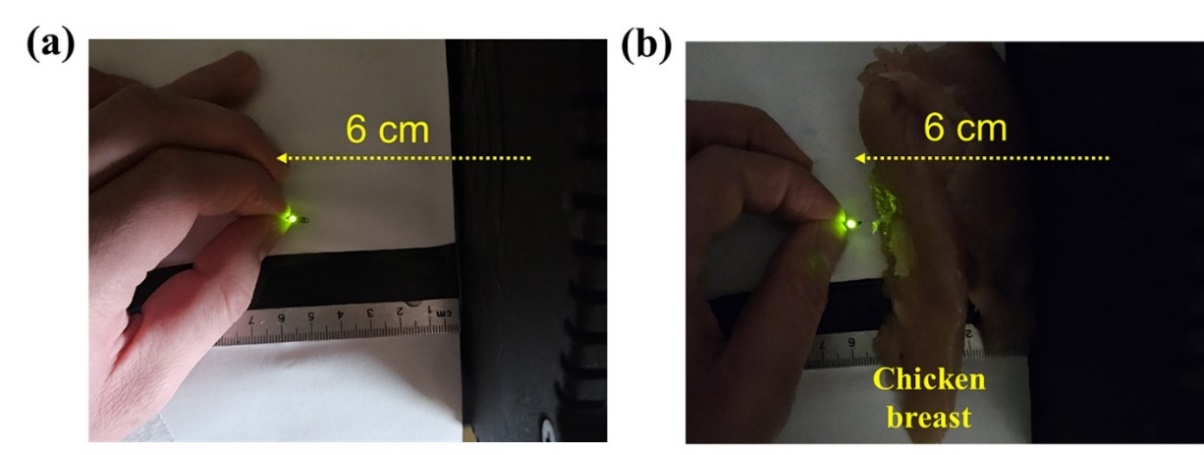

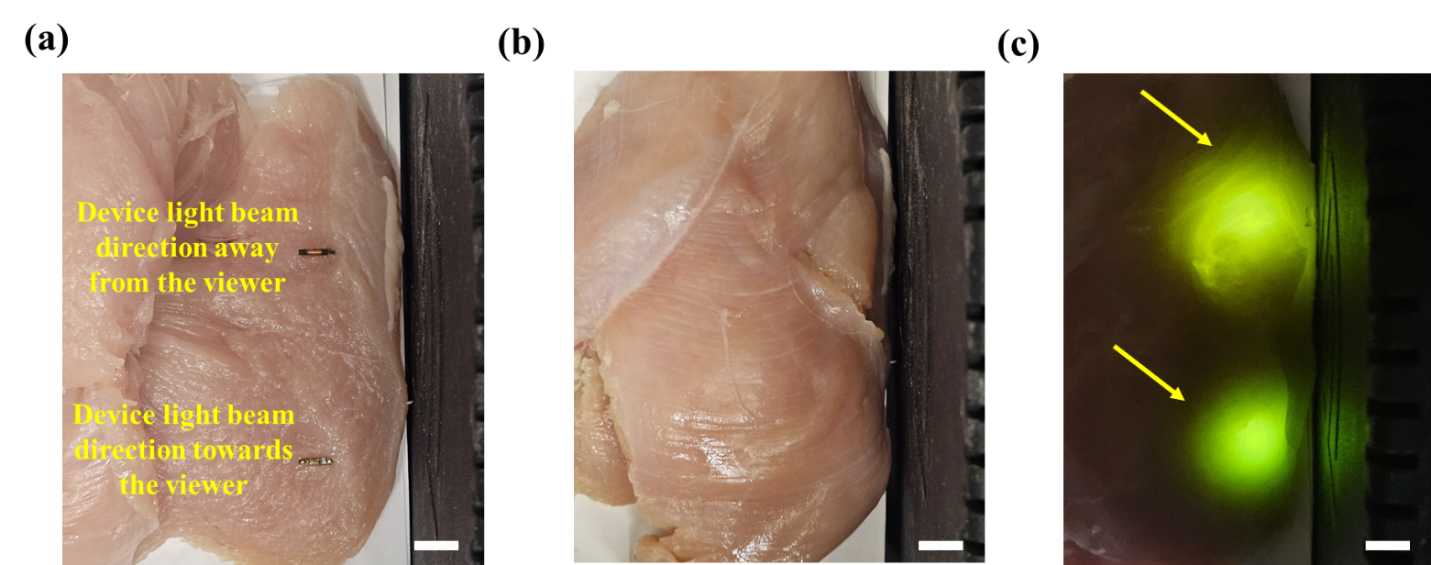


**Figure S3. Diffusion of device output light due to passage through chicken breast.** (a) The light beam from two devices is directed in opposite directions. (b) Photograph of chicken breast containing the two devices. (c) Photograph of the diffused output light from the two devices that are buried in the chicken breast (lab lighting is off). Scale bars = 10 mm.


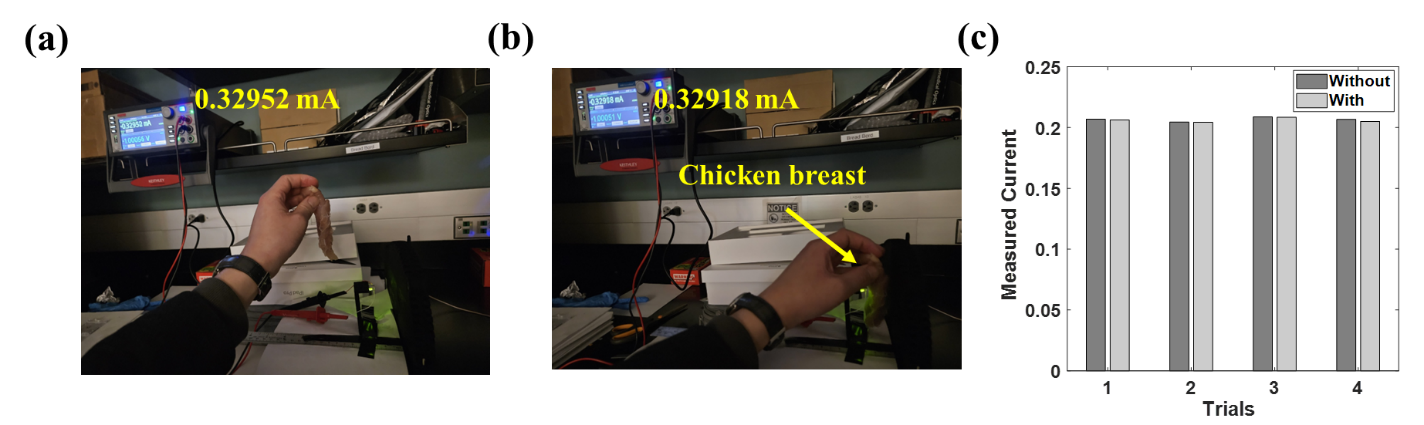
**Figure S4. Effect of chicken breast between device and antenna** (a) The device and antenna are separated by air. (b) The device and antenna are separated by chicken breast.

**Figure S5. Optical output measurement without and with chicken breast.** The optical output is measured without chicken breast (a) with chicken breast (b) by placing it between TX and device. The measured current at different distance for four times for each are presented.


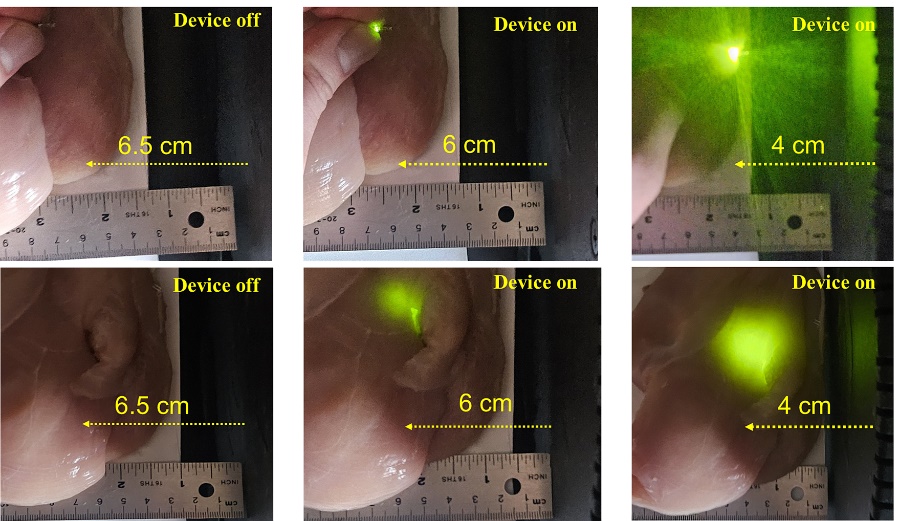


**Figure S6. Change in light output intensity with device-antenna distance in air vs device in chicken breast.** The top row shows photographs of the device outside the chicken breast with only air in the device-antenna space. The bottom row shows photographs with the device buried in chicken breast.

1. **DPBF Reaction with Singlet Oxygen**


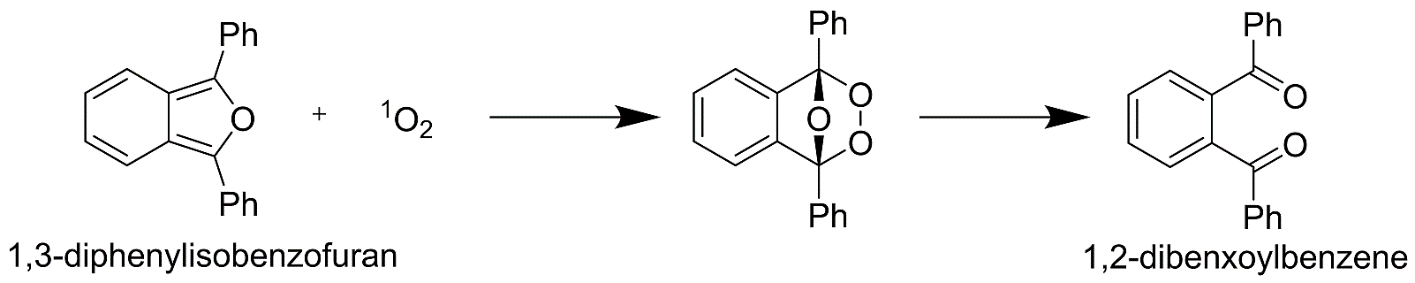


**Scheme S1.** The reaction scheme of 1,3-diphenylisobenzofuran (DPBF) with singlet oxygen to produce a colorless product.

1. **HT-29 Cell Culture Control Experiments**

**
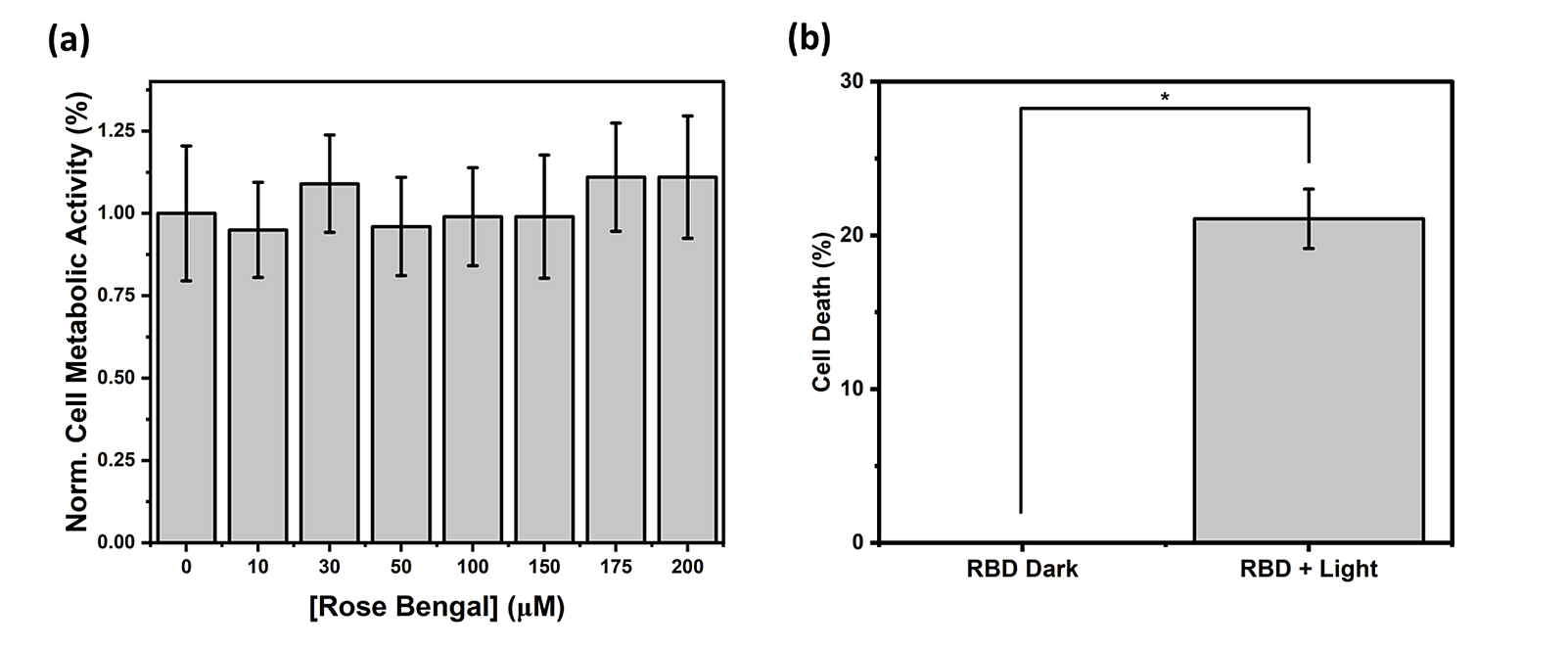
**

**
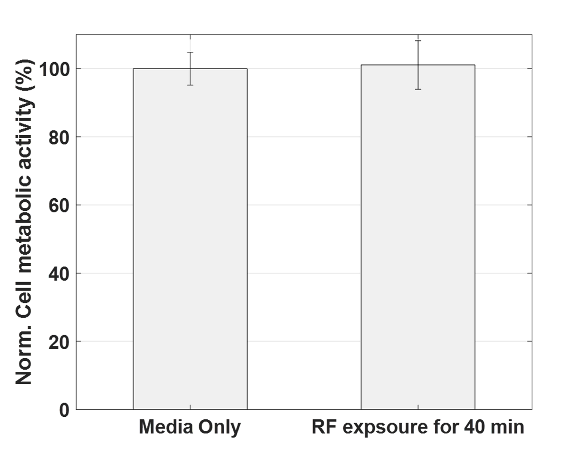
Figure S7.**  **Normalized metabolic activity (MTT assay) of HT-29 cells and LDH cytotoxicity.** (a) Normalized metabolic activity of HT-29 cells incubated in McCoy’s 5A media containing Rose Bengal (0 – 200 μM) where bars are an average of N = 3 for each concentration. (b) Percentage cell death measured by release of lactose dehydrogenase (LDH) from HT-29 cells incubated with Rose Bengal Diacetate (RBD; 30 μM) and left in the dark or irradiated (30 min) with the wireless device; bars are the average of N = 3 for each concentration. * p < 0.05

**Figure S8.** HT-29 cell metabolic activity (MTT assay) after RF exposure (6 W) at room temperature for 40 minutes in the dark. There was no difference in metabolic activity.

1. **Photoinactivation of HT-29 Cells with Rose Bengal Diacetate**


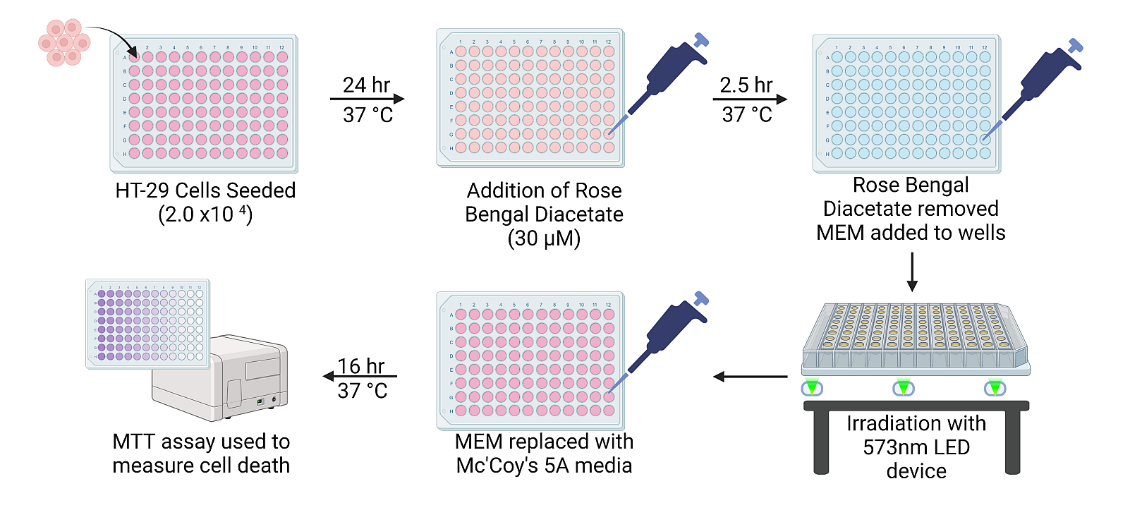


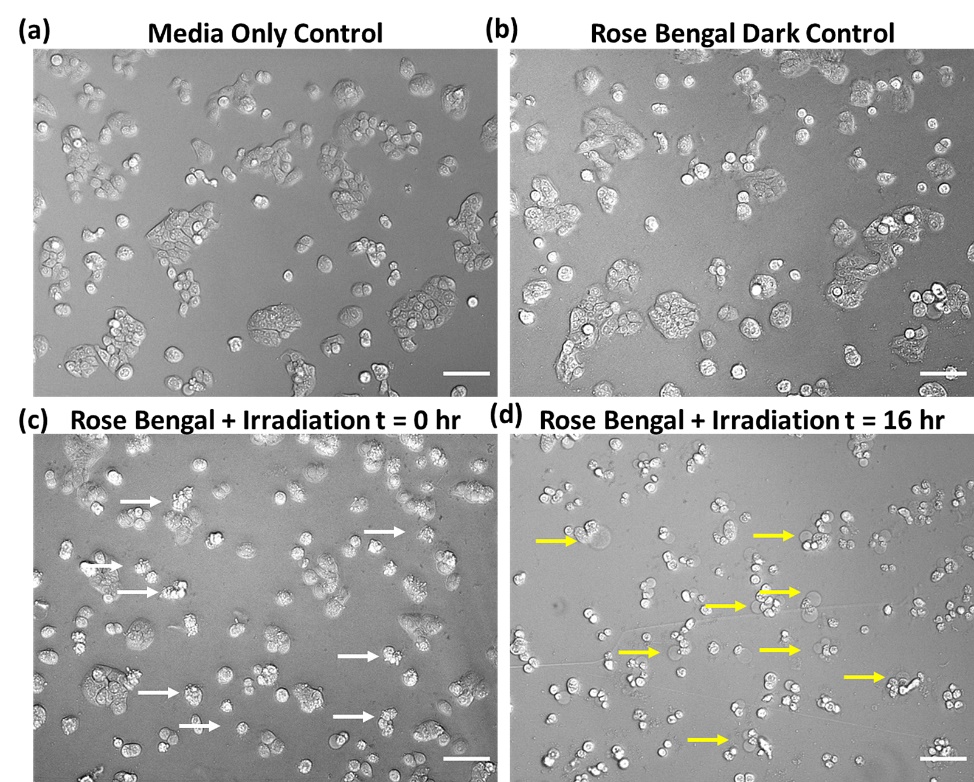
**Scheme S2:** Workflow of HT-29 cell photosensitization experiment with miniature LED-based device. Figure made with Biorender.

**Figure S9. HT-29 cell morphology changes caused by photoinactivation.** Micrographs (20x) show, (a) Control cells, (b) Cells treated with Rose Bengal Diacetate (30 μM) and kept in the dark, (c) Cells treated with Rose Bengal Diacetate (30 μM), irradiated with the wireless device (30 minutes) imaged directly after irradiation, and (d) imaged 16 hours after irradiation. White arrows indicate membrane ruffling; yellow arrows indicate protruding bubble. Scale bar = 50 µm.


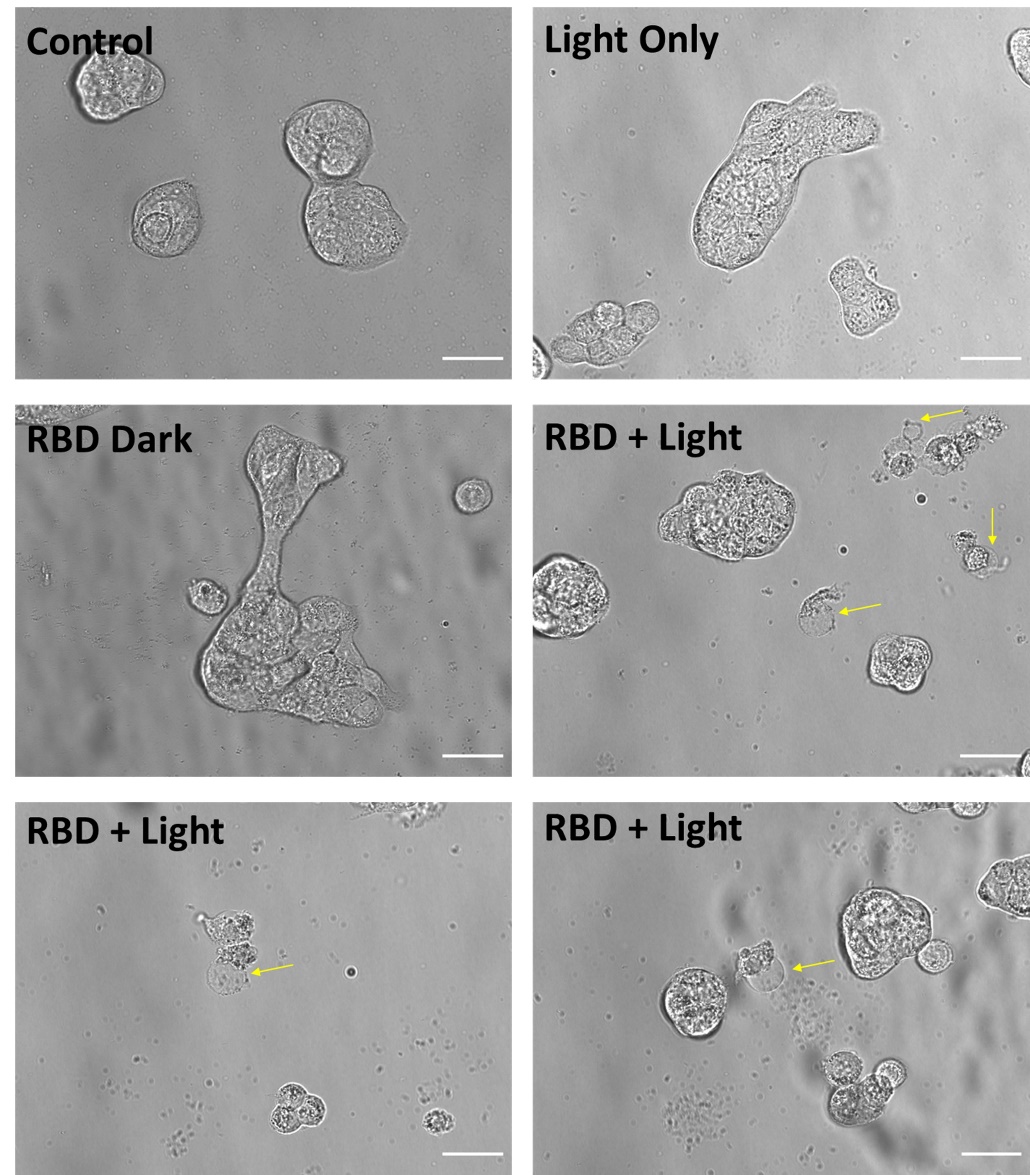


**Figure S10. Phase contrast imaging of HT-29 morphological changes.** Microscopy images. HT-29 cells (40x) were captured with oblique illumination and 1.5x digital zoom. Control: Untreated HT-29 cells. Light Only: Irradiation with the wireless device (30 min) and imaged 16 hours later. RBD Dark: Rose Bengal Diacetate (RBD) treated cells (30 μM) kept in the dark. RBD + Light: RBD treatment (30 μM), irradiation (30 min), and imaged 16 hours later. Yellow arrows indicate protruding bubbles. Scale bar = 30 µm


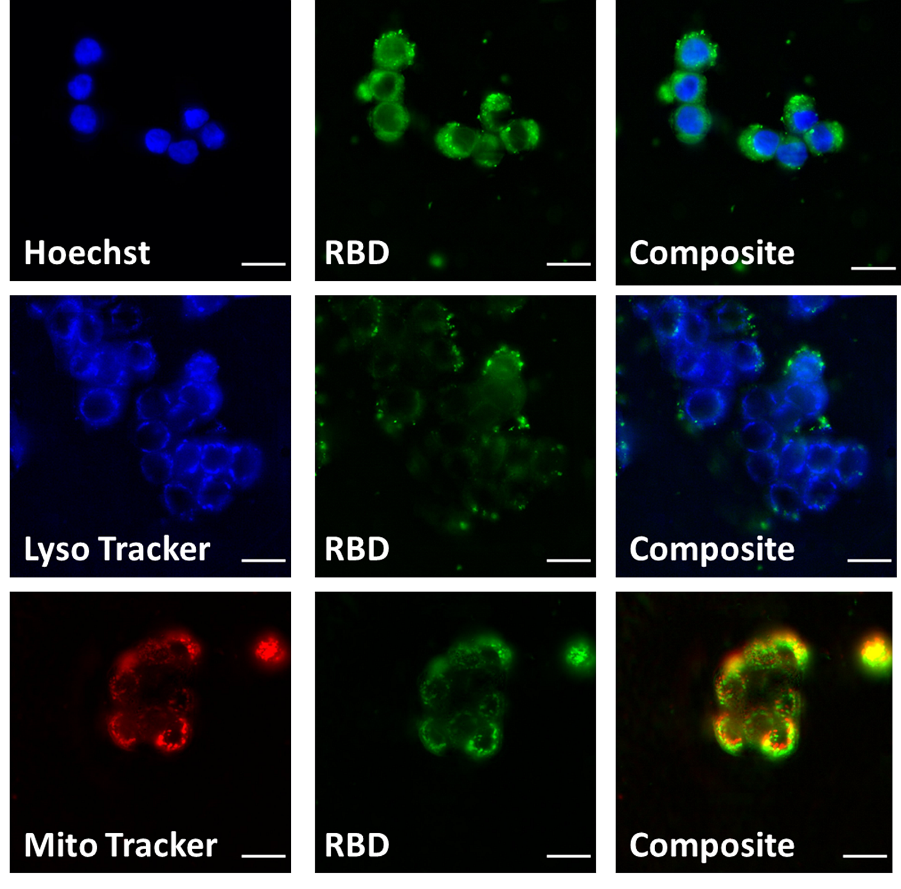


**Figure S11. Subcellular localization of Rose Bengal Diacetate.** Microscopy images. HT-29 cells (63x). Cells treated with Rose Bengal Diacetate (RBD; 30 µM) 2.5 hours before the addition of either Hoechst (3 µM), Lyso Tracker Blue DND-22 (150 nM), or Mito Tracker Deep Red (150 nM) and incubating for 10 minutes. RBD – TxRed (Ex: 562/40, Em 624/40), Hoechst and Lyso Tracker Blue DND-22 – DAPI (Ex: 387/11 nm, Em 447/60 nm), Mito Tracker Deep Red – Cy5.5 (Ex: 655/40 nm, Em 716/40 nm). Scale bar = 50 µm. There is co-localization of the RBD fluorescence only with the Mitotracker fluorescence.


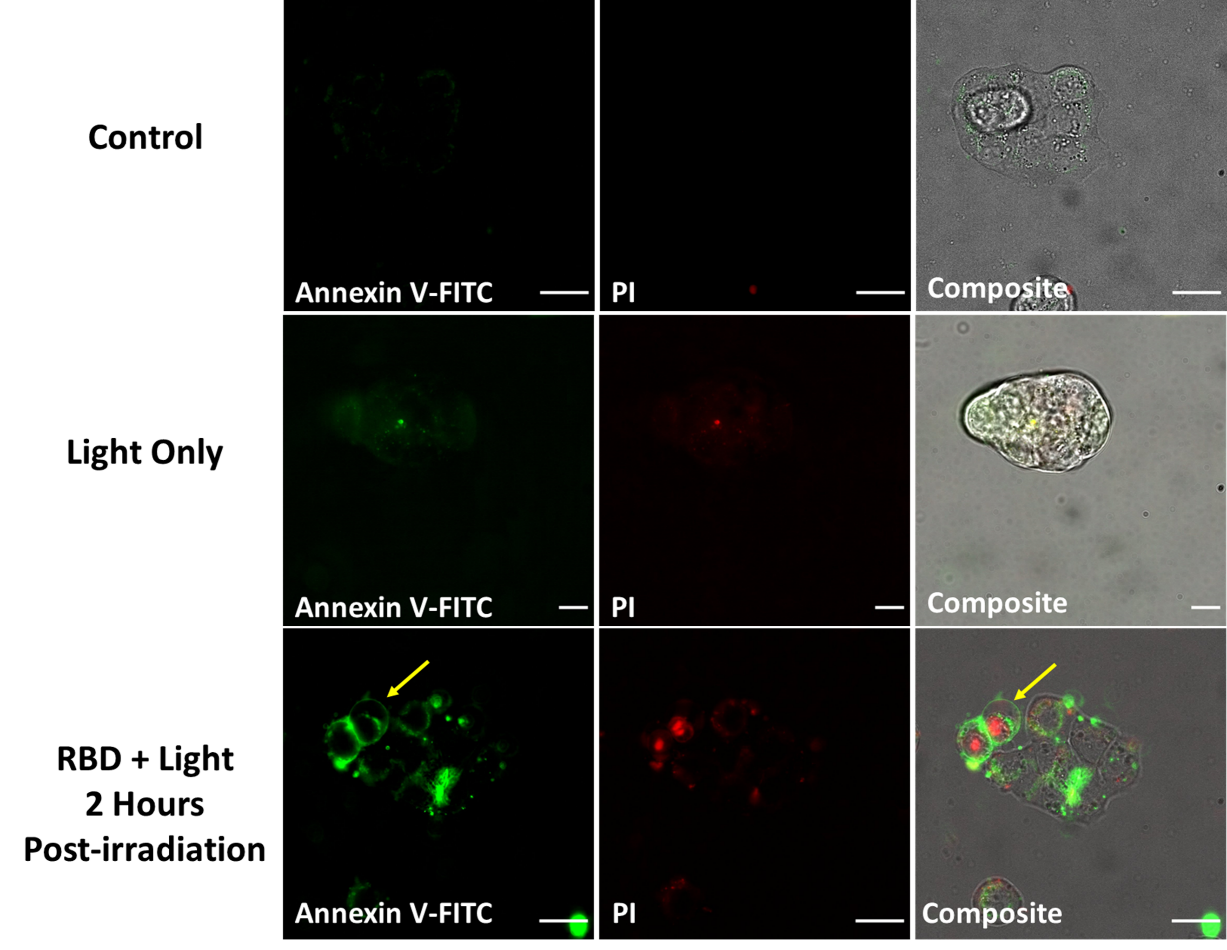


**Figure S12. Fluorescence microscopy of HT-29 cells.** Fluorescence micrographs (63x) show control cells (top row), cells after a 30 minute irradiation using wireless device and incubating two hours (middle row), cells treated with Rose Bengal Diacetate (30 µM) for 2.5 hours, after a 30 minute irradiation using wireless device, and waiting 2 hours (bottom row). Cells were stained with Annexin-V FITC (Ex: 485/20 nm, Em: 524/24 nm) and Propidium Iodide (PI) (Ex: 562/40 nm, Em 624/40 nm). Yellow arrow indicates a single protruding bubble (pyroptotic body). Scale bar = 50 µm


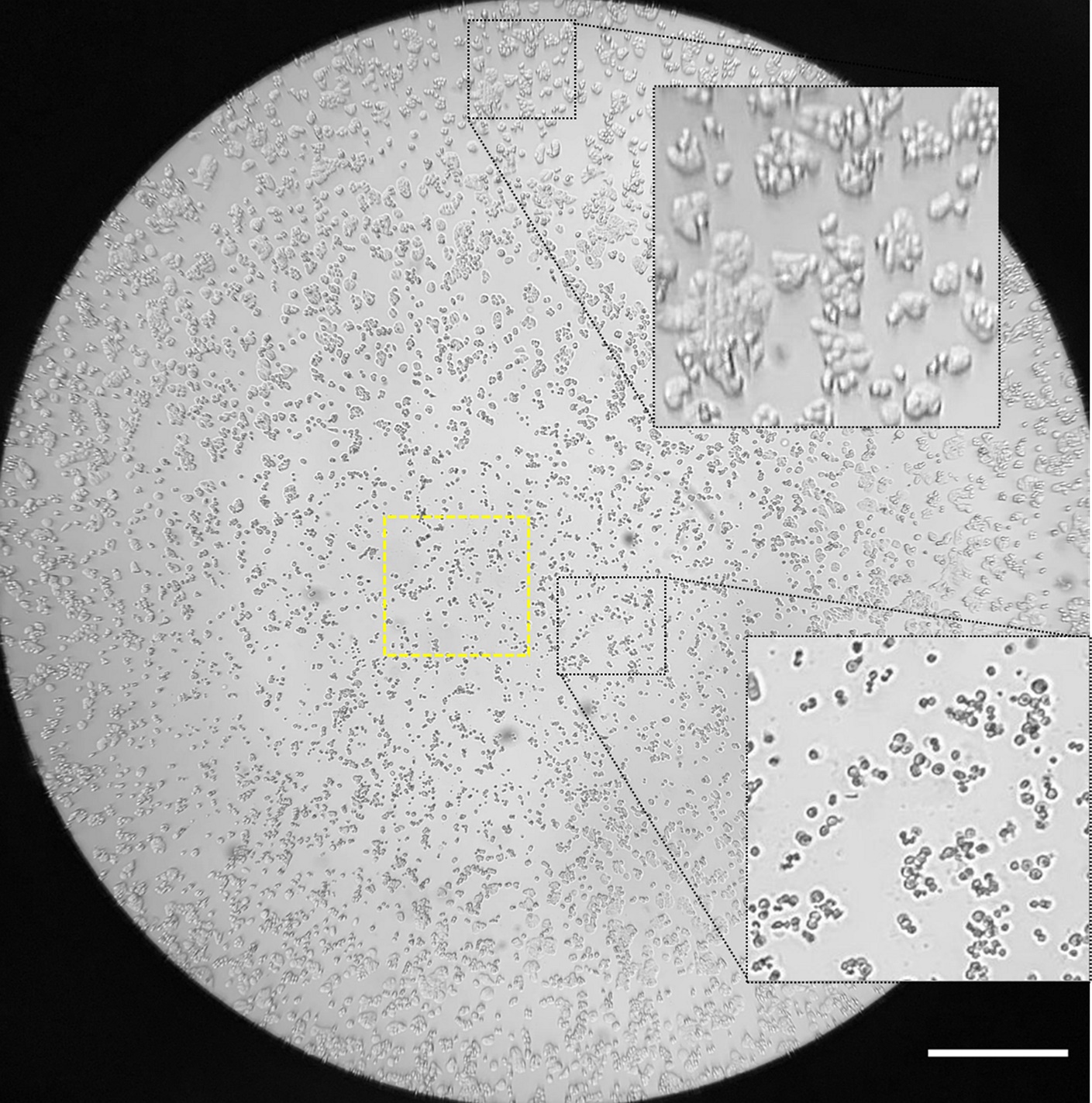


**Figure S13. Distance dependence on cell photoinactivation.** Micrograph (5x) of HT-29 cells treated with Rose Bengal Diacetate (30 μM) and irradiated with the wireless device for 30 minutes. The brightfield image was captured 16 hours post irradiation. The location of the device is outlined in yellow. The top “zoomed in” panel shows that cells distant from the irradiation exhibit healthy cell morphology. The bottom “zoomed in” panel shows that cells proximal to the irradiation site exhibit dead cell morphology. Scale bar = 1 mm.
